# *De Novo* Transcriptome Sequence Assembly and Analysis of RNA Silencing Genes of *Nicotiana benthamiana*

**DOI:** 10.1371/journal.pone.0059534

**Published:** 2013-03-28

**Authors:** Kenlee Nakasugi, Ross N. Crowhurst, Julia Bally, Craig C. Wood, Roger P. Hellens, Peter M. Waterhouse

**Affiliations:** 1 School of Molecular Bioscience, University of Sydney, Sydney, Australia; 2 Mount Albert Research Centre, Plant and Food Research, Auckland, New Zealand; 3 Commonwealth Scientific and Industrial Research Organisation–Plant Industry, Canberra, Australia; University of Leeds, United Kingdom

## Abstract

**Background:**

*Nicotiana benthamiana* has been widely used for transient gene expression assays and as a model plant in the study of plant-microbe interactions, lipid engineering and RNA silencing pathways. Assembling the sequence of its transcriptome provides information that, in conjunction with the genome sequence, will facilitate gaining insight into the plant’s capacity for high-level transient transgene expression, generation of mobile gene silencing signals, and hyper-susceptibility to viral infection.

**Methodology/Results:**

RNA-seq libraries from 9 different tissues were deep sequenced and assembled, de novo, into a representation of the transcriptome. The assembly, of16GB of sequence, yielded 237,340 contigs, clustering into 119,014 transcripts (unigenes). Between 80 and 85% of reads from all tissues could be mapped back to the full transcriptome. Approximately 63% of the unigenes exhibited a match to the Solgenomics tomato predicted proteins database. Approximately 94% of the Solgenomics *N. benthamiana* unigene set (16,024 sequences) matched our unigene set (119,014 sequences). Using homology searches we identified 31 homologues that are involved in RNAi-associated pathways in *Arabidopsis thaliana*, and show that they possess the domains characteristic of these proteins. Of these genes, the RNA dependent RNA polymerase gene, Rdr1, is transcribed but has a 72 nt insertion in exon1 that would cause premature termination of translation. Dicer-like 3 (DCL3) appears to lack both the DEAD helicase motif and second dsRNA binding motif, and DCL2 and AGO4b have unexpectedly high levels of transcription.

**Conclusions:**

The assembled and annotated representation of the transcriptome and list of RNAi-associated sequences are accessible at www.benthgenome.com alongside a draft genome assembly. These genomic resources will be very useful for further study of the developmental, metabolic and defense pathways of *N. benthamiana* and in understanding the mechanisms behind the features which have made it such a well-used model plant.

## Introduction

*Nicotiana benthamiana* is a native tobacco that grows in isolated communities in remote regions of northern Australia, ranging from Western Australia to Queensland. It is an allo-tetraploid with 19 chromosome pairs and has 26 exclusively Australian native tobacco relatives [1]. An isolate of *N. benthamiana* from a single collection appears to have been passed from lab to lab over the last 80 years [2] and has been used widely in the study of plant-microbe interactions. *N. benthamiana* has been particularly useful to plant virologists as it is susceptible to over 500 different viruses [3] and more recently as a host for Virus Induced Gene Silencing (VIGS). VIGS technology is based on infecting plants with a virus that has been engineered to contain a fragment of the target plant gene. As the virus replicates and spreads the plant’s RNA interference mechanism generates short interfering RNAs (siRNAs) from the engineered viral genome, including the inserted fragment, and with these target silencing of the intended gene. This approach has helped to elucidate the functions of many different plant genes [4]. Over the last fifteen years, *N. benthamiana* has been increasingly adopted by the plant molecular biology community as the host for transient transgene expression by Agroinfiltration. In this method, *Agrobacterium tumefaciens,* containing a transgene construct, is infiltrated into the leaf using a needle-less syringe. The cells of the leaf are transiently transformed and the transgene, from within the T-DNA borders, is highly and rapidly expressed. This system has been particularly useful for examining the functions of proteins, their intracellular localization [3], [5], [6], as a design tool for metabolic engineering [7], [8] and for functional confirmation of forward and reverse genetics discoveries made in *A. thaliana* [9].

In addition to its role in VIGS, *N. benthamiana* has played a major part in characterisation of RNA silencing processes. It has become the species of choice in which to visualise silencing and silencing suppression, using GFP reporter transgenes. This includes showing the interactions between the plants’ silencing system and viral suppressors [10], [11], and the propagation and spread of mobile RNA silencing signals. However, almost all of genes in the plants’ RNA silencing pathways have been discovered and characterised in *Arabidopsis*. With this regard the visualisation and study of the processes in *N. benthamiana* has mainly relied on over-expression of *Arabidopsis* genes or constructs using *N. benthamiana* gene fragments that have been amplified from the genome using primers designed on partial ESTs or sequences from heterologous species.

To provide a better foundation upon which to use *N. benthamiana* as a model plant, we have assembled transcript sequences from 9 tissue types to provide a representation of the *N. benthamiana* transcriptome. This should enhance the annotation and use of the draft genome sequences of *N. benthamiana* [7], [12]. We have also focused on identifying genes involved in the RNA silencing and associated pathways, and provide an overview of their relative abundances in the different tissues.

## Materials and Methods

### Tissues and Total RNA Isolation

*Nicotiana benthamiana* plants were grown at 21°C under a 16-h photo-period and an 8-h dark period in an environmentally controlled glasshouse. Apex, capsule, flower, leaf, roots, seedling and stem samples from 6-week old plants were collected and immediately frozen in liquid nitrogen before storage at −80°C until further RNA extraction. For plants submitted to drought stress the water supply was stopped one week before the collection of the leaves (herein called Dstress). Tissues culture samples were grown from sterile *N. benthamiana* leaf pieces sub-cultured on MSN media. Green calli, approximately 4 weeks old, were collected and stored in the same conditions as the other samples (herein called tissue culture (TC)).

Total RNA was isolated from 500 mg of the selected plant tissues using the CTAB RNA extraction method [13]. Briefly, 5 ml of preheated (65°C) total RNA extraction buffer (2% (w/v) CTAB (Sigma), 2% (w/v) polyvinylpyrrolidone (PVP-40) (Sigma), 100 mM Tris HCl (pH 8.0), 25 mM EDTA (pH 8.0), 2 M NaCl and 2% β-mercaptoethanol) was added to each sample grounded in liquid nitrogen. Each sample was then extracted twice with an equal volume of Chloroform: Isoamylalcohol (24∶1), mixed and centrifuged at 4560 g for 20 minutes at room temperature. The resulting supernatant was carefully transferred into a new tube, mixed with LiCl at a final concentration of 2 M and incubated overnight at 4°C. After the incubation the samples were centrifuged at 4000 g for 20 min at 4°C and the resulting pellets were dissolved in 500 µl of preheated SSTE buffer (1 M NaCI, 0.5% SDS, 10 mM Tris HCl (pH 8.0), and 1 mM EDTA (pH 8.0)), extracted again with an equal volume of Chloroform: Isoamylalcohol and then washed with 75% ethanol and vacuum dried. Dried RNA pellets were diluted in RNAse free sterile water and stored at −80°C until used. The integrity of total RNA was determined by running samples on 1% denaturing agarose gel. The concentration and quality was initially assessed using a spectrophotometer (NanoDrop, Technologies Inc.) at an absorbance ratio of A260/230 and A260/280 nm. A Bioanalyzer (Agilent) was used to perform a final assessment on the quality prior to deep sequencing.

### Deep Sequencing

RNA-seq libraries for each tissue were prepared from total RNA using the Illumina Truseq v1 RNA sample prep protocol (not strand specific) at the Australian Genome Research Facility (service provider). Briefly, poly-A mRNA were purified using oligo (dT) primed magnetic beads and chemically fragmented into smaller pieces. Cleaved fragments were converted to double-stranded cDNA using random hexamer primers. After purification and end-repair, the cDNA fragments were ligated to Illumina paired-end sequencing adapters which contain sample specific indexes to allow for sequencing of multiple libraries on a single lane of an Illumina flowcell. Following this, fragments were purified, size selected on a gel, and then amplified by PCR to obtain the final library. The libraries representing 9 tissues were then pooled in equal quantities into a single aliquot for sequencing on the Illumina HiSeq-2000 platform at the Australian Genome Research Facility, according to the manufacturers’ protocols. The libraries were sequenced as paired-end 100 nt reads. After sequencing, the reads were pre-processed as described below. All reads have been deposited in the Sequence Read Archive (SRA) at NCBI, under accession number SRA066161.

### *De novo* Transcriptome Assembly, Annotation and Assessment of Completeness

Deep sequencing reads were quality assessed with the quality assessment software FastQC (http://www.bioinformatics.babraham.ac.uk/projects/fastqc/). For every tissue sample, reads were quality filtered (Q25) and trimmed (12 nt from 5′ end, and 5 nt from 3′ end) with the FastX-toolkit software suite (http://hannonlab.cshl.edu/fastx_toolkit/). The quality filtering of paired-end reads results in some reads losing its partner read. Further processing to extract properly-paired reads and orphaned (singleton) reads was performed with a script from the SHRIMP package [14].

All post-processed reads from the 9 tissue samples were pooled together and assembled *de novo* with Abyss v1.3 [15] with k-mer sizes ranging from 58 to 80 with a step size of 2. The k-mer assemblies were then merged with Trans-Abyss v1.1 [16]. Computational and storage resources were provided by Intersect Australia Ltd (http://www.intersect.org.au), and each k-mer assembly was allowed 16 CPUs and 60 GB of RAM. Each assembly typically completed within 24 hours.

*De novo* transcripts were annotated using Plant and Food Researchs’ in-house BioView Sequence Analysis and Annotation pipeline. BioView annotated the *de novo* transcripts by searching the SwissProt [17], Uniref90 (http://www.uniprot.org/downloads) [18], RefSeq (release 54) [19], and *A. thaliana* proteins (TAIR10) (http://www.arabidopsis.org/) databases using BLASTx (version 2.2.25) [20]. Searching against the NCBI non-redundant (NR) DNA database (ftp://ftp.ncbi.nlm.nih.gov/BLAST/db/) was performed using BLASTn (version 2.2.25) [20]. InterPro and Gene Ontology (GO) terms were derived following motif searching using InterProScan (version 4.8) [21] and InterPro Release 38 (http://www.ebi.ac.uk/interpro/). Each transcript was given an ID such as Nbv3K745626388, which depicts the assembly version (Nbv3), k-mer size from which the transcript was assembled (K74), and a 7 digit ID assigned by BioView.

The final “description” for each *de novo* transcript was based on a series of searches as well as the ratio lengths to the HSP (highest-scoring segment pairs) from BLAST. For each transcript, the best description and BLAST metrics for the best HSP to SwissProt was taken. The process was repeated with UniRef90 if no SwissProt matches were found, repeated with RefSeq if no UniRef matches were found, repeated with TAIR if no RefSeq matches were found, and repeated with the non-redundant (NR) databases in NCBI. Next, the HSP to sequence length ratios for query and subject was determined (qRatio = HSP length as percentage of query length; sRatio = HSP length as percentage of subject length). If the qRatio and sRatio are > = 90% and the description contains any of hypothetical, unknown, unclassified or uncharacterised terms, then the description is set to “highly conserved hypothetical protein”. If the qRatio and sRatio are > = 90% and identity is greater or equal to 50% but less than 90%, then it is called a “conserved hypothetical protein” if the description contains hypothetical, unknown, unclassified or uncharacterised terms. Otherwise “putative” is appended to the description. If the qRatio and sRatio are less than 90% but above 50% then “similar to” is appended. Otherwise “probable” is appended unless it is hypothetical, in which case the transcript is annotated a “hypothetical protein”.

For the creation of a unigene set of the raw assembly, cd-hit-est from the CD-HIT package [22] with a identity parameter of 95% was utilised. This will subsume all shorter sequences into a longer transcript if they have a sequence identity of 95% or greater.

To assess the completeness of the transcriptome assembly, the CEGMA (Core Eukaryotic Genes Mapping Approach) software was applied to identify the presence of a core protein set consisting of 248 highly conserved proteins that are found in a wide range of eukaryotes [23]. This software is usually used to assess the completeness of a genome assembly, but should also enable the assessment of a transcriptome under different interpretations. The transcriptome assembly was also compared to the tomato (*Solanum lycopersicum*) predicted proteins database (SL2.4) and the *N. benthamiana* unigene dataset v1 build from the Solgenomics database (http://solgenomics.net) using BLAST (threshold E-value of 1e^−3^).

Mapping statistics were obtained using standalone Bowtie v0.12.8 [24], mimicking parameters used in RSEM, except for the reporting of unique and multi-mapping reads (the options ‘-a -m 1’ were used).

The transcriptome assembly with annotations can be downloaded as a fasta file at http://www.benthgenome.com.

### Transcript Abundance Analyses

Transcript quantification of the *de novo* assembly was carried out with RSEM, which allows for an assessment of transcript abundances based on the mapping of RNA-seq reads to the assembled transcriptome [25]. Briefly, RSEM calculates maximum likelihood abundance estimates as well as posterior mean estimates and 95% credibility intervals for genes/isoforms. Abundance estimates are generated in the form of two measures, one which gives an estimate of the number of fragments that can be derived from an isoform or gene (the expected counts (EC)), and the other which is the estimated fraction of transcripts within the sample that is represented by the given isoform or gene.

To adjust for library sizes and skewed expression of transcripts, the EC values were normalized using the Trimmed Mean of M-values (TMM) normalization method [26] utilising an edgeR script from the Trinity package [27], [28]. This method calculates the effective library size of each sample which was then used to normalize the EC values (FPKM (Fragments Per Kilobase per Million) values were not calculated). The second metric from RSEM was multiplied by one million to obtain a measure given as transcripts per million (TPM). To assess the distribution of the EC and TPM values for each tissue, basic statistics and residuals (the difference between a data point and the median) were calculated and plotted with the statistics package, R [29]. The TPM value is preferred over other metrics such as FPKM [30] and RPKM (Reads Per Kilobase per Million) [31] as it is independent of the mean expressed transcript length and thus more comparable between different species and samples [25]. Due to the nature of how EC and TPM measures are derived and applied [25], [26], [28], [32], [33], for comparisons of abundance TPM values were used, whereas other comparisons made use of normalized EC values.

### Identification of RNAi Genes

Thirty one well-characterized RNAi associated genes (RNAi genes from here on) from *A. thaliana* (Table S4) were used to search for homologues in *N. benthamiana.* Protein sequences were retrieved from TAIR, and first screened against the tomato predicted protein database (SL2.40) at Solgenomics using BLASTp [34]. Both *A. thaliana* and tomato RNAi protein sequences were then used to screen the transcriptome assembly of *N. benthamiana* using tBLASTn. The most significant matches to *A. thaliana* and tomato queries were manually assessed and in cases of multiple matches, the query coverage (%) and identity (%) of high-scoring segment pairs were analysed further. Where possible, a full length sequence was manually reconstituted from the shorter transcript sequences, and where this was not possible, sequence data from our draft genome assembly [7] and/or from the *N. benthamiana* sequences deposited in the Solgenomics database [12] was used. All putative transcripts were subsequently translated and reciprocally checked against the TAIR and tomato databases to compare and confirm their identities. Domain searches of the translated sequences of the RNAi genes was performed with InterProScan (http://www.ebi.ac.uk/Tools/pfa/iprscan/), using all the default databases. An E-value of 1e^−3^ was used as the cut-off threshold, and Pfam results were given precedence. Abundances of the transcripts constituting an RNAi gene are reported as TPM values as described above.

The bioinformatically identified *N. benthamiana* RNAi CDS and protein sequences can be downloaded at http://www.benthgenome.com.

### Phylogeny Analysis

Sequences of homologous RNAi proteins from *Oryza sativa* (http://rice.plantbiology.msu.edu/), *Populas trichocarpa* (http://www.phytozome.net/poplar), tomato, *A. thaliana*, *Nicotiana attenuata*, *Nicotiana tabacum*, *Nicotiana glutinosa,* and previously reported *N. benthamiana* entries (all *Nicotiana* species were retrieved from NCBI Genbank), were retrieved for phylogeny analyses. Accession numbers are reported in the figures. Alignments and tree construction were performed in the Geneious Pro version program (http://www.geneious.com), using the Muscle algorithm for alignments, and Neighbour Joining method with bootstrapping of 1000 for consensus tree construction.

### Confirmation of an Insertion Sequence in Rdr1 in *N. benthamiana*

To confirm the insertion sequence in Rdr1 in *N. benthamiana*, PCR was carried out on a 1,035 nt region containing the insertion, using the forward primer 5′-CACCATGCAAAGTTTATTTTTCTGGTCCAG and reverse prime 5′-GCCCGGAAAGTTTGCAGCATCATTGAAAGA. These primers were also used to amplify the region from another commonly used *N. benthamiana* variant, 16C. The region was also amplified from *N. tabacum*. All sequences were subcloned and determined using conventional BigDye 3.1 chemistry.

## Results and Discussion

### *De novo* Transcriptome Assembly

The sequences of the *N. benthamiana* transcriptome were captured by preparing RNA-seq libraries from seven different tissues grown under normal conditions (apex, capsule, flower, leaf, roots, seedling and stem), and from two samples of tissues under stress (drought stressed leaf and tissue culture callus). To counter the inherent sequencing biases incurred in RNA-seq library preparation such as a reduced 5′ sequence complexity in the reads which could affect the assembly ([35]–[39], http://seqanswers.com/forums/showthread.php?t=11843), and to maximise the quality, the pooled 368,674,918 raw 100 nt reads from the libraries were trimmed by 12 nt and 5 nt at their 5′ and 3′ends, respectively, and reads containing bases below a quality score of 25 were discarded. This resulted in a total of 197,872,501 (76,224,365 paired-end and 45,423,771 singleton reads) post-processed 83 nt RNA-seq reads that were used for *de novo* transcriptome assembly. This very stringent filtering gave high quality sequences, but at the cost of discarding approximately 50% of the raw reads. Abyss v1.3 [15] was used to make assemblies from the reads, using increasing k-mer sizes from 58 to 80 (step size of 2), and the k-mer assemblies were merged using Trans-Abyss [16].

A summary of the assembly statistics is given in Table 1, and shows that the complete assembly yielded 237,340 contigs with a median contig size of 510 nt and a maximum contig size of 7969 nt. The total size of the assembly is ∼188 Mb with an average of about 56-fold coverage. The raw assembly contigs were clustered into a unigene dataset, using a threshold nucleotide identity of 95%, to produce 119,014 contigs (a reduction of ∼50%) with a total size of 89.6 Mb (Table 1). This is substantially more than the recently reported 73,041 unigenes (total size of 37.8 Mb) from a fungus-infected *N. benthamiana* transcriptome obtained using a Velvet/Oases assembly [40]. However, the Abyss/Trans-Abyss assembly pipeline tends to generate a large total number of contigs with a high proportion of them under 500 nt, especially from polyploid plant transcriptomes [41]–[43], and 50% of the unigenes in our assembly are between 100 and 500 nt (Table 1). Nonetheless, Trans-Abyss is reported to generate a better overall representation of transcripts over a broad range of expression levels [16], [41].

**Table 1:** Statistics of the *de novo* transcriptome assembly.

Total number of input reads (as singletons)	197872501	
Paired	76224365	
Orphaned	45423771	
Read length	83	
	**Full**	**Unigenes**
Total size of contigs	1.88E+08	89550506
Number of contigs	237340	119014
Mean contig size	794	752
Median contig size	510	486
Min contig size	81	109
Max contig size	7969	7969
Number of contigs >100 nt	237335	119014
Number of contigs >500 nt	119944	58396
Number of contigs >1000 nt	67089	30076

The sizes of the libraries used for the assembly are given in Table 2. While the number of reads from each tissue varied, especially for seedling, approximately 80 to 85% of paired reads from each tissue were able to map back to the assembled transcriptome. As also discussed later, the contribution of reads to the assembly from all tissues appeared to be quite similar.

**Table 2:** Mapping statistics of reads from each tissue to the transcriptome assembly.

	Apex	Capsule	Dstress	Flower	Leaf	Root	Seedling	Stem	TC
Total reads	7982949	16946882	13511245	13904896	15492665	14801396	90081852	14188920	10961696
Number of singleton reads	1794295	3942278	2956639	3215352	3570565	3421770	20764812	3237574	2520486
Number of properly paired reads	3094327	6502302	5277303	5344772	5961050	5689813	34658520	5475673	4220605
**Properly paired read mappings to all contigs**									
Unique mapping pairs	636942	1333015	1068272	1187332	1181070	1183725	6843574	1086467	889774
Multi-mapping pairs	1846302	4047038	3160791	3198648	3718383	3681321	22002639	3377221	2627857
Un-mappable pairs	611083	1122249	1048240	958792	1061597	824767	5812307	1011985	702974
Percentage of mappable reads in library	80.25	82.74	80.14	82.06	82.19	85.50	83.23	81.52	83.34
**Properly paired read mappings to >500** **nt contigs**									
Unique mapping pairs	658098	1380468	1079509	1204381	1192679	1237355	6845002	1102866	902369
Multi-mapping pairs	1668747	3786276	2975615	2888572	3392882	3441306	19113437	3150478	2442318
Un-mappable pairs	767482	1335558	1222179	1251819	1375489	1011152	8700081	1222329	875918
Percentage of mappable reads in library	75.20	79.46	76.84	76.58	76.93	82.23	74.90	77.68	79.25
**Properly paired read mappings to >1000** **nt contigs**									
Unique mapping pairs	552755	1417269	1041322	1045083	1157024	1111401	6319347	1060379	841743
Multi-mapping pairs	1133912	2848170	2349818	2208774	2586029	2549677	12603350	2474767	1868513
Un-mappable pairs	1407660	2236863	1886163	2090915	2217997	2028735	15735823	1940527	1510349
Percentage of mappable reads in library	54.51	65.60	64.26	60.88	62.79	64.34	54.60	64.56	64.21

During our search for RNAi genes (described below), we observed that sequence variants of some genes showed a high nucleotide sequence identity (∼95%), and solely using the unigene transcript set would not have led to the identification and reconstitution of putative full length CDS sequences. We therefore considered both the raw transcriptome assembly and unigene dataset, where relevant, for subsequent analyses.

### Assessment of Assembly

The quality and completeness of our *N. benthamiana* transcriptome assembly was assessed in three different ways: using CEGMA, by comparison with publicly available *N. benthamiana* sequences, and by comparison with tomato sequences from Solgenomics.

The CEGMA software [23] can be used to assess the completeness of a transcriptome assembly by evaluating the presence and completeness of a widely conserved set of 248 eukaryotic proteins, as has been applied elsewhere [40], [44]. These proteins are mostly from housekeeping genes and therefore can be expected to be expressed [45]. Analysis of our raw transcriptome assembly identified 236 out of the 248 core proteins (95%) as ‘complete’ (defined as >70%, alignment length with core protein). In addition, there was an average of ∼4 orthologues per core protein, with 219 of those detected having more than 1 orthologue. Repeating the analysis on the unigene dataset detected 237 core proteins, with an average of ∼3 orthologues per core protein and 194 having more than 1 orthologue. Compared to *A. thaliana* which has on average 2 orthologues per core protein [23], *N. benthamiana* appears to have about 3 to 4 orthologues per core protein (based on transcriptome data). It is tempting to speculate that this is due to its allo-tetraploidy, but ancestral whole genome duplication and allelic variation are also likely events. It will be interesting to see if these results are consistent with a genomic assessment, the assemblies of which are still in draft stages [7], [12].

Comparing our *N. benthamiana* unigene set with the *N. benthamiana* unigenes from the Solgenomics database (total of 16,024 sequences as of November 2012, based on predictions from genomic sequences and ESTs) using BLASTn and an E-value filter of 1e^−3^, returned 15,039 (93.9%) matching to our unigene set, of which 14,826 (92.5%) have >90% sequence identity, and 14,166 (88.4%) have >95% sequence identity. Using sequence sizes between 100 and 500 nt, 501 and 1000 nt, and >1001 nt, gave 88.9%, 96.2% and 99.3% matching to our unigene dataset, respectively. The GC content of both datasets was approximately 41%.

The raw *N. benthamiana* transcriptome assembly was compared with the Solgenomics tomato genome predicted proteins database, using BLASTx (E-value <1e^−3^), and showed that 69,429 out of 237,340 transcripts (29.3%) have a match with a sequence identity greater than 90%, while 152,838 (64.4%) match with a sequence identity greater than 70%. In total, 174,421 (73.5%) exhibited a match to the tomato protein database. For the unigene dataset, a total of 74,492 out of 119,014 transcripts (62.6%) matched against the tomato protein database. This is in comparison to 43.8% and 85.4% of unigenes from other reported transcriptomes of *N. benthamiana* and *N. tabacum*, respectively, matching to the same tomato protein database [40], [46].

Taken altogether, these three analyses suggest that our *N. benthamiana* protein-coding transcriptome is a broad representation of the plant’s gene expression potential and that, while the *N. benthamiana* homologues of tomato proteins are identifiable, there is quite some amino acid sequence diversity between the counterparts in the two species.

### Read Mapping to the *N. benthamiana* and Tomato Genomes

When the RNA-seq reads from each library were mapped back to the tomato and the two available *N. benthamiana* draft genomes [7], [12], [47], only up to 5% of reads could map back to the tomato genome compared to approximately 75% of all reads that could map back to the two versions of the *N. benthamiana* genomes (Table 3). While the metrics reported here are dependent on the mapping parameters (e.g. read length, seed length, number of mismatches allowed), the low read mapping percentage reflects a high nucleotide sequence divergence between tomato and *N. benthamiana.* It appears that there is a 1 in 12 base difference in gene-rich regions between the tomato and potato genomes [47], and given that tomato and potato are much more closely related, at least in the transcript space [48], the low mapping percentage of our RNA-seq reads to the tomato genome is not unexpected. Interestingly, there was a higher percentage of unique reads mapping to our draft *N. benthamiana* genome [7] compared to the one available in the Solgenomics database [12], although the overall proportions was very similar. This is likely due to differences in the ‘completeness’ of the assemblies, and perhaps some nucleotide differences accumulated by different lines passed down in different laboratories.

**Table 3:** Percentage of total reads from 9 tissues that mapped uniquely and to multiple locations on the tomato (Solgenomics, SL2.40) and two versions of the draft *N. benthamiana* genomes.

	*N. benthamiana* (ANZ consortium)			*N. benthamiana* (Solgenomics)			Tomato		
	% unique	% multi	% of total	% unique	% multi	% of total	% unique	% multi	% of total
Apex	51.52	23.33	74.85	46.88	26.70	73.58	1.30	0.43	1.73
Capsule	53.88	22.55	76.43	49.19	25.84	75.03	1.08	0.21	1.30
Dstress	56.63	20.04	76.67	53.03	22.19	75.21	1.04	0.22	1.26
Flower	50.98	25.95	76.93	46.64	28.91	75.56	2.70	2.20	4.90
Leaf	50.97	25.36	76.33	46.91	27.72	74.64	2.09	1.69	3.78
Root	52.14	23.44	75.58	47.93	26.46	74.39	1.31	0.24	1.55
Seedling	49.47	25.24	74.71	43.87	29.32	73.20	1.34	0.24	1.58
Stem	51.52	23.92	75.44	47.33	26.62	73.95	1.47	0.30	1.77
TC	51.72	23.64	75.36	47.45	26.41	73.87	1.33	0.28	1.61

### Annotation

**Figure 1:**
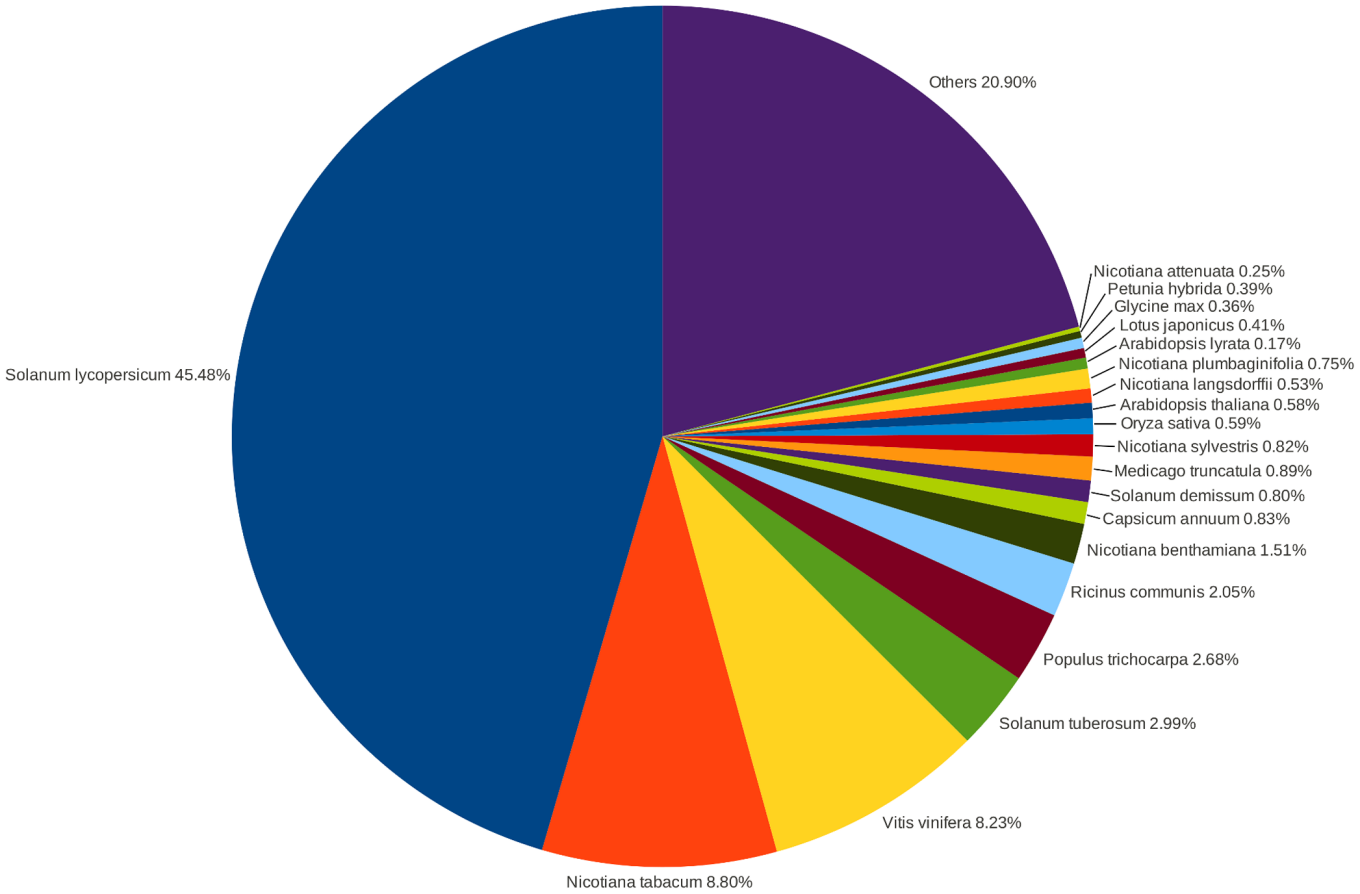
Species distribution of the transcriptome assembly. A BLASTx using a cutoff value of E-value <1e^−3^ was used to search the transcriptome assembly against Genbank’s non-redundant database.

The assembled *N. benthamiana* transcriptome was annotated from comparisons with entries described in SwissProt, RefSeq, UniProt, TAIR, and Genbank databases. The proportion of the 119,014 unigenes showing matches with records in these databases ranged from 41.2% with SwissProt, to 68.8% with Genbank (Table 4). Not unexpectedly, the matches between the raw transcriptome and entries in Genbank’s NR protein database showed 45.5% of transcripts having significant similarity to tomato sequences, followed by 8.8% with *N. tabacum* (Figure 1). The species with the next most hits (8.2%) was *Vitis vinifera* (Grape seed), followed by smaller percentages of hits with other members of the Solanaceae. Clearly, the number of hits, *per se*, is not an absolute measure of relatedness between the species but rather a composite of the relatedness and the scope of available sequence data. This is well illustrated by only 1.5% of transcripts matching other available *N. benthamiana* entries, reflecting the prior lack of *N. benthamiana* sequences.

**Table 4:** Number of matches from complete transcriptome (237,340 transcripts) and unigene set (119,014 transcripts) to five databases.

	SwissProt	RefSeq plant	Uniprot	TAIR proteins	Genbank
Total matches to database	204575	214605	208399	224225	237271
Unique matches (E-value <1e−3)	122003	166440	166216	155596	185243
Percentage of all transcripts	51.40	70.13	70.03	65.56	78.05
Unique unigene matches (E-value <1e−3)	49013	66484	68473	64051	81920
Percentage of all unigenes	41.18	55.86	57.53	53.82	68.83

Gene ontology (GO) terms could be assigned to 41,016 (17.3%) of the 237,340 raw transcripts and 16,169 (13.6%) of the 119,014 unigene transcripts. This is comparable to the 15.3% of 95,916 unigenes annotated with GO terms in *N. tabacum* [49]. The *N. benthamiana* unigene transcripts were further refined to GO slim terms, annotating 25.5% as having a biological process (GO:008150), 24.3% to being a cellular component (GO:005575), and 24.3% to having a molecular function (GO:0003674). The distribution of the unigenes into GO slim categories is provided in Figure S1.

**Figure 2:**
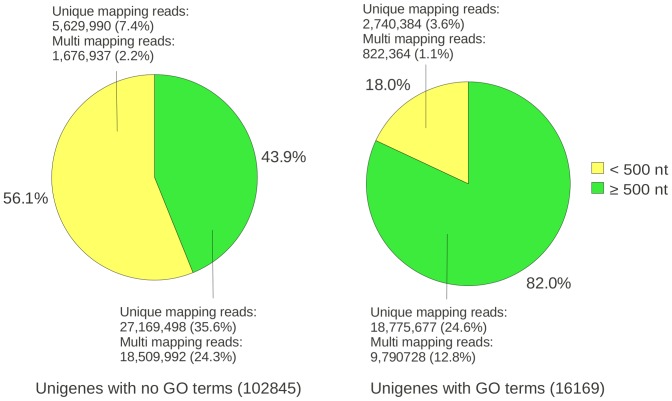
Proportion of unigenes with and without GO annotations. The figure shows the percentage of unigenes larger (green) and smaller (yellow) than 500 nt that could be annotated with (right) or without (left) GO terms. Mapping metrics show the number of uniquely and multi-mapping reads as a proportion of total number of reads used in the transcriptome assembly. For unigenes without GO terms, the total number of bases of transcripts under and over 500 nt was 14,954,024 nt and 54,714,622 nt, respectively. For unigenes assigned GO terms, the total number of bases under and over 500 nt was 890,086 nt and 18,991,774 nt, respectively.

To better understand why only such a relatively small proportion of unigenes could be annotated with GO terms, the transcriptome mapping statistics were examined. This revealed that 82% of the GO-assignable unigene transcripts were >500 nt in length and 56% of the GO-unassignable transcripts were in the <500 nt size range (Figure 2). Moreover, read mapping statistics indicated that coverage was 30-fold lower for GO-unassignable transcripts that were <500 nt in size compared to those >500 nt in length (Figure 2). This showed that a high proportion of the assembly is comprised of short transcripts that make a relatively small contribution to the protein-coding transcriptome, and could be the representation of lowly expressed genes and/or from high-level transcription of non-coding RNAs. Such observations mirror transcriptome assembly studies of other polyploid plants, which also report high percentages of unassigned transcripts [40], [46], [50], [51].

### Transcript Abundance in Individual Tissues and Mapping Statistics

**Figure 3:**
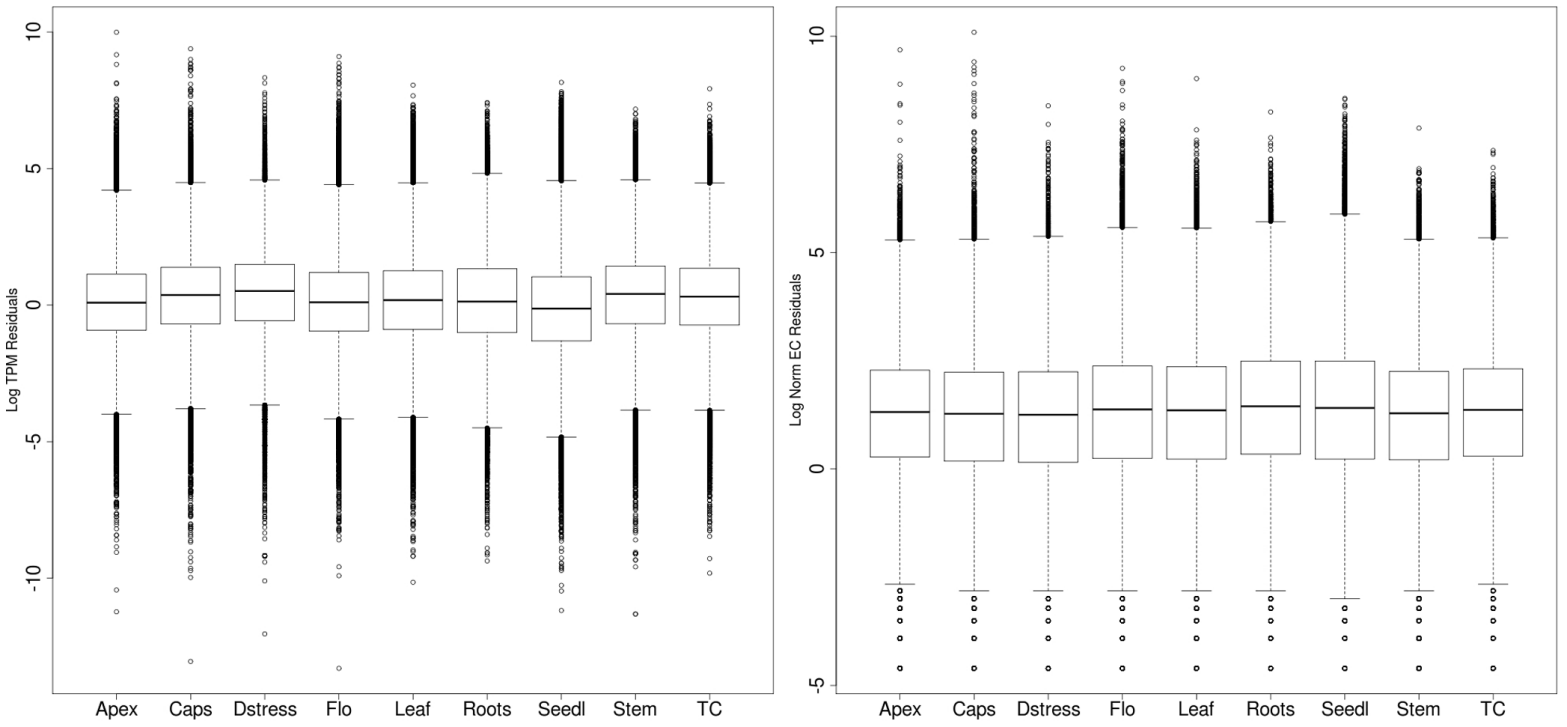
Distribution of TPM and EC residuals. Boxplots of the residuals (see methods) of TPM (Transcripts Per Million) (left) and normalized EC (Expected Counts) (right) values in different tissues showing the distribution of these values about the median. For the right panel, only EC values greater than 0 were used.

**Figure 4:**
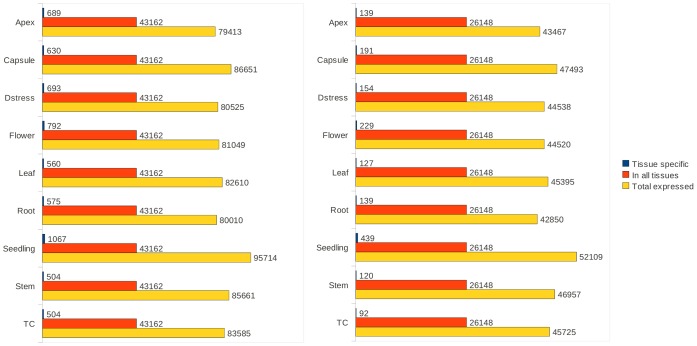
Number of transcripts detected in the 9 tissues. The figure shows the number of tissue-specific transcripts, and transcripts that were detected in all 9 tissues, that are over 500 nt in the transcriptome assembly. Left panel: number of transcripts from the full assembly. Right panel: number of transcripts from the unigene transcript set. Only transcripts with EC (Expected Counts) values of more than zero were used.

The representation of transcripts from each of the 9 tissues in the assembled transcriptome was evaluated in terms of expected counts (EC) and transcripts per million (TPM) generated by the RSEM software. For all tissues, the variation about the median was more uniform for TPM than for EC values, but overall the transcript expression profiles and the contribution of read data towards the assembly from each of the tissues were very similar (Figure 3). The median TPM values across all tissues ranged from 0.47 to 1.74, while the median for normalized ECs ranged between 1.13 and 1.98 (Table S1). However, a small proportion of transcripts appeared to be very highly expressed as shown by the difference in the 3rd quartile and maximum EC and TPM values for each tissue (Table S1). The different tissues had a common set of about 26,000 unigene transcripts that were greater than 500 nt but each tissue also uniquely expressed a number of transcripts (Figure 4). The undifferentiated callus cells grown in tissue culture produced the fewest transcripts exclusive to that tissue (92 transcripts) whereas the sample with the most unique transcripts came from seedlings (439 transcripts). This abundance of unique transcripts in seedlings may reflect the extra diversity of proteins required for rapid growth and differentiation, however, it may also be a measure of the depth of reads that were obtained for the sample, because its RNA-seq library had at least 5 times more reads than any other sample. Nonetheless, the proportion of all mappable reads (unique and multi-mapping) was similar for all tissues (Table 2), and taken together with the assessment of the distribution of transcript abundance values, the contribution of reads to the assembly does not appear to be skewed to any one tissue.

Each tissue had a similar GO category distribution of expressed transcripts (Table S2), but the most highly expressed transcripts varied according to tissue type (Table 5 and Table S3). The apex and flower tissues had a prevalence of transcripts encoding defence related genes while the transcripts in seedling, leaf, and stem tissues were predominantly from photosynthesis and cell structure genes. The most highly expressed transcripts in capsules, which include developing seeds, were for seed storage proteins (globulins) and, unsurprisingly, the most highly expressed transcripts in the drought stressed leaves were from genes associated with stress.

**Table 5:** Top 10 transcripts expressed transcripts (based on TPM values) in the 9 tissues used forthe transcriptome assembly.

	TPM	Description		TPM	Description
**Apex**			**Root**		
Nbv3K765635181	21909.14	Protein TAP1 precursor	Nbv3K765637499	1669.75	Phosphatidylserine decarboxylase beta chain
Nbv3K765636475	9619.12	Defensin-like protein, Precursor	Nbv3K805659615	1632.48	Tubulin alpha chain
Nbv3K805655645	6757.63	Ethylene-responsive proteinase inhibitor 1,	Nbv3K605746981	1510.15	Putative lipid-transfer protein DIR1, Precursor
		Precursor			
Nbv3K725839522	3424.63	Wound-induced proteinase inhibitor 2, Precursor	Nbv3K805655878	1229.00	MLP-like protein 43
Nbv3K785653169	3318.90	Non-specific lipid-transfer protein (LTP),	Nbv3K785643634	1218.77	Tubulin alpha-2 chain
		Precursor			
Nbv3K785643755	1899.95	hypothetical protein	Nbv3K805664031	1190.27	Fruit-specific protein
Nbv3K785644141	1894.17	Organ-specific protein S2	Nbv3K605748986	1135.91	Glycine-rich protein, Precursor
Nbv3K725616268	1756.37	defensin-like protein	Nbv3K605747902	1105.61	Glucan endo-1,3-beta-glucosidase, basic vacuolar isoform GGIB50 ((1->3)-beta-glucanase), Precursor
Nbv3K765632833	1519.04	serine protease inhibitor 2	Nbv3K725841908	1097.43	Foot protein 1 variant 1, Precursor
Nbv3K805664026	1492.69	hypothetical protein	Nbv3K765632492	1093.49	Metallothionein-like protein type 2
**Capsule**			**Seedling**		
Nbv3K805662064	11957.53	11S globulin seed storage protein 2 basic chain,	Nbv3K805662530	3493.49	Ribulose bisphosphate carboxylase small chain
		Precursor			8B, chloroplastic (RuBisCO small subunit 8B),
					Precursor
Nbv3K785648979	8103.38	Legumin B basic chain, Precursor	Nbv3K805661563	2485.08	Oxygen-evolving enhancer protein 3-2,
					chloroplastic (OEE3), Precursor
Nbv3K805658849	6989.87	11S globulin seed storage protein G3 basic chain,	Nbv3K805660593	2351.66	Vesicular integral-membrane protein VIP36,
		Precursor			Precursor
Nbv3K705821994	6752.20	2S albumin large chain, Precursor	Nbv3K725839270	2297.67	Ribulose bisphosphate carboxylase small chain
					8B, chloroplastic, Precursor
Nbv3K785649564	6163.69	Legumin B basic chain, Precursor	Nbv3K805660696	2274.73	Ferredoxin-1, chloroplastic, Precursor
Nbv3K785654438	5910.78	11S globulin seed storage protein G3 basic chain,	Nbv3K785653197	2172.34	Ribulose bisphosphate carboxylase small chain
		Precursor			8B, chloroplastic, Precursor
Nbv3K805657565	5520.25	Glutelin type-B 4 basic chain, Precursor	Nbv3K805658276	2041.68	chlorophyll a-b binding protein
Nbv3K705825953	5461.75	11S globulin delta chain, Precursor	Nbv3K805656982	2027.77	chlorophyll a-b binding protein
Nbv3K685810384	5417.70	2S albumin large chain, Precursor	Nbv3K785643703	1958.04	Chlorophyll a-b binding protein CP29.1,
					chloroplastic, Precursor
Nbv3K625771317	4444.27	Legumin B basic chain, Precursor	Nbv3K805658200	1952.76	chlorophyll a-b binding protein
**Dstress**			**Stem**		
Nbv3K805657258	4158.01	Vignain, Precursor	Nbv3K805659615	1313.56	Tubulin alpha chain
Nbv3K805662978	3399.45	E3 ubiquitin ligase interacting with arginine	Nbv3K785653169	1105.28	Non-specific lipid-transfer protein (LTP), Precursor
		methyltransferase (ISS)			
Nbv3K785647202	2390.34	Kirola	Nbv3K785643634	1101.55	Tubulin alpha-2 chain
Nbv3K785646208	2241.37	Retrotransposon-derived protein PEG10 (MyEF-3)	Nbv3K805662029	916.63	Putative uncharacterized protein ART2
Nbv3K785647827	1959.69	Vignain, Precursor	Nbv3K805660095	868.53	Tubulin alpha chain
Nbv3K805661569	1726.16	Abscisic acid and environmental stress	Nbv3K785651507	827.10	hypothetical protein
		inducible protein TAS14			
Nbv3K725841908	1557.96	Foot protein 1 variant 1, Precursor	Nbv3K805664610	798.36	Thiazole biosynthetic enzyme, chloroplastic,
					Precursor
Nbv3K745620329	1495.50	hypothetical protein	Nbv3K805658269	781.03	Metallothionein-like protein type 2
Nbv3K785651507	1340.51	hypothetical protein	Nbv3K805663773	770.90	Metallothionein-like protein type 2 B
Nbv3K745627524	1319.71	Male-specific sperm protein Mst84Dc	Nbv3K805656914	764.39	Ribulose bisphosphate carboxylase/oxygenase
					activase 1, chloroplastic (RA 1), Precursor
**Flower**			**TC**		
Nbv3K805662029	8996.18	Putative uncharacterized protein ART2	Nbv3K805655878	2769.66	MLP-like protein 43
Nbv3K805658722	7047.10	Heme A synthase (HAS)	Nbv3K805663311	1571.49	Kirola
Nbv3K805664066	6114.56	Neurophysin IT 1, Precursor	Nbv3K785644141	1329.37	Organ-specific protein S2
Nbv3K805656915	5895.86	TRAF2 and NCK-interacting protein kinase	Nbv3K805664026	1002.15	hypothetical protein
Nbv3K805663996	5179.72	RRNA intron-encoded homing endonuclease	Nbv3K785645902	867.50	Probable aquaporin PIP-type pTOM75 (RAMP)
Nbv3K805663381	4649.96	Laminin subunit beta-1, Precursor	Nbv3K805661363	842.94	Non-specific lipid-transfer protein 1 (LTP 1),
					Precursor
Nbv3K705822805	4617.65	Regulator of rDNA transcription protein 15	Nbv3K605747902	828.55	Glucan endo-1,3-beta-glucosidase, basic
					vacuolar isoform GGIB50 ((1->3)-beta-
					glucanase), Precursor
Nbv3K805662548	4026.96	Protein YLR162W	Nbv3K785647691	816.97	hypothetical protein
Nbv3K805661757	3965.65	Putative uncharacterized protein YLR154W-F	Nbv3K785643634	763.45	Tubulin alpha-2 chain
Nbv3K705831290	3536.86	Aconitate hydratase	Nbv3K805662530	748.55	Ribulose bisphosphate carboxylase small chain
					8B, chloroplastic, Precursor
**Leaf**					
Nbv3K805662029	3158.40	Putative uncharacterized protein ART2			
Nbv3K805656915	2137.05	TRAF2 and NCK-interacting protein kinase			
Nbv3K805663381	1551.93	Laminin subunit beta-1, Precursor			
Nbv3K705822805	1497.99	Regulator of rDNA transcription protein 15			
Nbv3K805663773	1402.41	Metallothionein-like protein type 2 B			
Nbv3K805662548	1367.96	Protein YLR162W			
Nbv3K705831290	1350.36	Aconitate hydratase			
Nbv3K805658722	1329.29	Heme A synthase (HAS)			
Nbv3K785653197	1203.91	Ribulose bisphosphate carboxylase small chain			
		8B, chloroplastic, Precursor			
Nbv3K805663996	1194.86	RRNA intron-encoded homing endonuclease			

The *A. thaliana* genome has recently been shown to have unsuspected transcriptome complexity from alternative splicing. A total of 57,447 transcripts representing 23,901 genes were detected by RNA-seq, and more than 60% of the genes containing introns displayed some form of alternative splicing [52]. Our unigene set of 119,014 *N. benthamiana* transcripts (Table 1) is possibly an overestimate. However, there are 76,379 predicted transcripts in the draft *N. benthamiana* genome in the Solgenomics database (http://solgenomics.net/tools/BLAST/dbinfo.pl), and the number of unigenes of a fungus-infected *N. benthamiana* was reported to be 73,041 [40]. It will be interesting to see how important and widespread differential splicing is in this allopolyploid transcriptome.

### Identification of RNA Silencing Genes and Family Members

**Figure 5:**
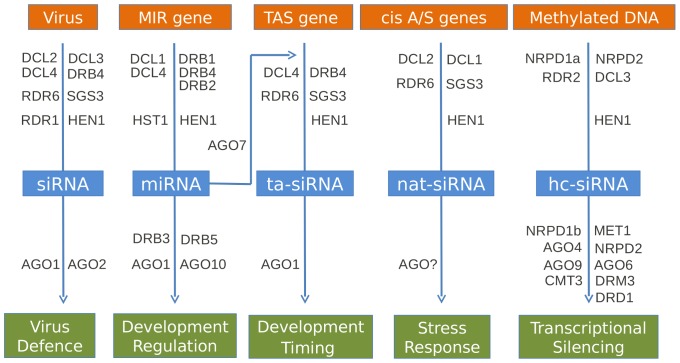
RNAi-associated pathways and their core components in plants. The schematic depicts the major proteins involved in producing different small RNAs (blue boxes) from RNA transcripts of various templates (orange boxes) and using these sRNAs to regulate a spectrum of important biological processes (green boxes).

The gene silencing (including RNAi) machinery in plants generates small 21–24 nt RNAs and uses them to guide at least five interwoven pathways providing different forms of gene regulation and plant defence (Figure 5). The core components of the pathways are encoded by gene families of the dicer-like (Dcl) nucleases, argonaute (Ago) proteins, double stranded RNA binding (Drb) proteins, RNA polymerases and methyl-transferases. In association with other proteins, they give targeted RNA degradation, translational repression and heterochromatin modification [53]. Many of the effects of these processes have been studied in *N. benthamiana* but with little detail about the intrinsic pathway components and assuming that they are similar to those characterised in *A. thaliana*. We investigated the presence and characteristics of RNAi gene transcripts in our *N. benthamiana* transcriptome assembly to gain a better insight into whether the plant may be defective or unusual in some of these pathways, as has been suggested [54], [55], and also as another way to evaluate the transcriptome assembly’s ‘completeness’.

Using the protein sequences of 31 well characterised *A. thaliana* RNAi genes (Figure 5 and Table S4) we first scanned for homologues in the Solgenomics tomato (*S. lycopersicum*) predicted proteins database. Screening the *N. benthamiana* transcriptome with the *A. thaliana* query set, aided by the *S. lycopersicum* counterparts, identified homologues for each RNAi gene query (Table 6) except for AGO3 (returned as AGO2) and AGO9 (returned as a AGO4 variant). Of the identified homologues, 24 of them appeared to be fully assembled. The AGO1, AGO2, AGO6, AGO7, DCL1, NRPD1a, NRPD1b, NRPD2a and RDR2 queries resulted in multiple hits due to the possible presence of additional gene family members or to shorter than full-length contigs (Table 6). The inability of the assembler to integrate these smaller contigs into full-length CDS transcripts is probably due to the presence of nucleotide polymorphisms from different mRNA splice-forms or from close family members, sequencing errors, and/or low sequencing depth. However with manual intervention, full-length CDS homologues of all but one were generated (Table 6). This was greatly assisted by the *N. benthamiana* sequences having both high sequence identities and high query coverage (>90%) with their *S. lycopersicum* counterparts. The one remaining partial assembly was of Nrpd1a. However, a 1414 aa sequence was compiled which, although shorter than its homologue in *A. thaliana* (by an estimated ∼30 aa), is much longer than the 1206 aa ORF in *S. lycopersicum*. The identified sequences were also compared to other *N. benthamiana* RNAi gene entries from Genbank and showed almost perfect identity (Figure S2).

**Table 6:** List of RNAi associated genes identified and compared to tomato and *A. thaliana* counterparts.

RNAi gene	Status	A.A. Length	Transcriptome Ids	Tomato ID; % identity to tomato; Query coverage	Athan ID; % identity to Athan; Query coverage
Ago1a	Full	1058	Nbv3K705826800	Solyc06g072300.2.1; 92.8%; 100%	AT1G48410.1 (AGO1); 82.7%; 89.3%
Ago1b*	Full	1110	Nbv3K785652119;	Solyc03g098280.2.1; 92.3%; 84.3%	AT1G48410.1 (AGO1); 81.3%; 85.9%
			Nbv3K785652117;		
			Nbv3K705830082		
Ago2*	Full	1093	Nbv3K585706870;	Solyc02g069260.2.1; 72.9%; 94%	AT1G31280.1 (AGO2); 50.1%; 84.4%
			Nbv3K585676062		
Ago4a	Full	912	Nbv3K585737054	Solyc06g073540.2.1; 94.4%; 100%	AT2G27040.1 (AGO4); 74.3%; 96.4%
Ago4b	Full	905	Nbv3K745626388	Solyc01g008960.2.1; 92.5%; 100%	AT2G27040.1 (AGO4); 74.6%; 96.7%
					AT5G21150.1 (AGO9); 69.9%; 97.6%
Ago5	Full	1013	Nbv3K585731374	Solyc06g074730.2.1; 67%; 95.1%	AT2G27880.1 (AGO5); 59.5%; 92.6%
Ago6*	Full	903	Nbv3K705827462;	Solyc07g049500.2.1; 86.4%; 100%	AT2G32940.1 (AGO6); 61.8%; 97.2%
			Nbv3K645784811;		
			Nbv3K645784061;		
			Nbv3K585708242		
Ago7*?	Full	991	Nbv3K585720936;	Solyc01g010970.2.1; 84.7%; 100%	AT1G69440.1 (AGO7); 68.2%; 87.4%
			Nbv3K585735851;		
			Nbv3K585719994;		
			Nbv3K665804949		
Ago10	Full	988	Nbv3K585734208	Solyc09g082830.2.1; 93.2%; 100%	AT5G43810.1 (AGO10); 80.6%; 100%
Cmt3a	Full	855	Nbv3K625768297	Solyc01g006100.2.1; 70.9%; 100%	AT1G69770.1 (CMT3); 54.8%; 89.8%
Cmt3b	Full	907	Nbv3K585704505	Solyc12g100330.1.1; 80.3%; 99.7%	AT1G69770.1 (CMT3); 54.9%; 85.1%
Dcl1*	Full	1909	Nbv3K605750463;	Solyc10g005130.2.1; 87.1%; 100%	AT1G01040.1 (DCL1); 71.6%; 100%
			Nbv3K745628138;		
			Nbv3K585722110		
Dcl2	Full	1402	Nbv3K725833766	Solyc06g048960.2.1; 83%; 98.3%	AT3G03300.1 (DCL2); 55.8%; 99.6%
Dcl3	Full	1456	Nbv3K585704110	Solyc08g067210.2.1; 83.5%; 94.9%	AT3G43920.1 (DCL3); 47%; 97.7%
Dcl4	Full	1622	Nbv3K625768999	Solyc07g005030.2.1; 85.9%; 94.6%	AT5G20320.1 (DCL4); 52.9%; 97.3%
Drb1	Full	316	Nbv3K605753726	Solyc04g076420.2.1; 61.8%; 94.3%	AT1G09700.1 (DRB1); 59.8%; 66.5%
Drb2a	Full	399	Nbv3K585718488	Solyc11g069460.1.1; 85.3%; 100%	AT2G28380.1 (DRB2); 64.7%; 87.5%
Drb2b	Full	400	Nbv3K605753084	Solyc11g069460.1.1; 85.5%; 100%	AT2G28380.1 (DRB2); 64.7%; 87.5%
Drb3	Full	469	Nbv3K725608215	Solyc05g056100.2.1; 80.8%; 97%	AT3G26932.1 (DRB3); 70.3%; 39.5%
					AT2G28380.1 (DRB2); 51.8%; 88%
					AT5G41070.1 (DRB5); 43.8%; 72.5%
Drb4	Full	352	Nbv3K725839976	Solyc01g056620.2.1; 64.7%; 99.7%	AT3G62800.1 (DRB4); 55.4%; 45.1%
Drb5	Full	477	Nbv3K585684100	Solyc05g056100.2.1; 71.6%; 96.9%	AT5G41070.1 (DRB5); 71.9%; 38.8%
					AT3G26932.1 (DRB3); 70.8%; 38.8%
					AT2G28380.1 (DRB2); 55%; 85.1%
Drd1	Full	926	Nbv3K585726931	Solyc01g109970.2.1; 84.8%; 98.6%	AT2G16390.1 (DRD1); 54.7%; 94.2%
Drm3	Full	705	Nbv3K745624408	Solyc05g053260.2.1; 82.3%; 76.5%	AT3G17310.2 (DRM3); 38.7%; 96.6%
Hen1	Full	948	Nbv3K645785075	Solyc05g026050.2.1; 82.5%; 99.6%	AT4G20910.1 (HEN1); 49.7%; 98.4%
Hst1	Full	1199	Nbv3K625768898	Solyc01g098170.2.1; 91.3%; 100%	AT3G05040.1 (HST); 65.5%; 98.1%
Met1	Full	1558	Nbv3K585692501	Solyc11g030600.2.1; 87.2%; 99.6%	AT5G49160.1 (MET1); 60.4%; 95.8%
Nrpd1a*	Partial	1414	Nbv3K585708997;	Solyc08g080210.2.1; 81.4%; 84.7%	AT1G63020.1 (NRPD1a); 48.6%; 98.2%
			Nbv3K585738556;		
			Nbv3K585698167		
Nrpd1b*	Full	2047	Nbv3K685818512;	Solyc01g096390.2.1; 86.1%; 72.1%	AT2G40030.1 (NRPD1b); 51.9%; 80%
			Nbv3K685816071;		
			Nbv3K585729430;		
			Nbv3K585720083		
Nrpd2a*	Full	1212	Nbv3K585720667;	Solyc03g110880.2.1; 92.8%; 100%	AT3G23780.1 (NRPD2a); 64.7%; 98.4%
			Nbv3K685813559		
Rdr1	Full	1140	Nbv3K745620210	Solyc05g007510.2.1; 83.1%; 99.9%	AT1G14790.1 (RDR1); 61.4%; 98.3%
Rdr2?	Full	1120	Nbv3K625766705	Solyc03g114140.2.1; 87%; 99.7%	AT4G11130.1 (RDR2); 59.1%; 99%
Rdr6	Full	1197	Nbv3K585707928	Solyc04g014870.2.1; 85%; 100%	AT3G49500.1 (RDR6); 65.3%; 100%
Sgs3	Full	635	Nbv3K785651293	Solyc04g025300.2.1; 81.2%; 99.8%	AT5G23570.1 (SGS3); 49.2%; 99.1%

Screening the *N. benthamiana* transcriptome assembly with AtAGO9 identified a potential counterpart transcript, Nbv3K745626388, but a reciprocal BLAST revealed it to be more similar to AtAGO4. However, scanning the *N. benthamiana* transcriptome with AtAGO4 had identified another transcript, Nbv3K585737054, as the most probable AGO4 homologue, and pair-wise comparison of these two *N. benthamiana* transcripts showed them to be 86.7% identical. From this we inferred that these two transcripts represent different Ago4 family members, which we designate Ago4a (Nbv3K585737054) and Ago4b (Nbv3K745626388). From a similar analysis, Ago1 also appears to have 2 gene family members (Table 6). These results confirm the previous identification of two variants of Ago1 and Ago4 in *N. benthamiana* [56]. Many of the other RNAi genes in *N. benthamiana* may also contain multiple family members. Multiple copies of several RNAi genes do exist in *S. lycopersicum* [57], and preliminary analyses of single nucleotide polymorphisms in our RNAi genes do suggest the presence of other members in some (data not shown).

In addition to our assessment of the assembly completeness, the identification of apparently full length RNAi gene homologues from 30 of 31 queries, the near to possibly full-length assembly of the remaining Nrpd1a homologue, and the identification of additional family members in Drb2, Ago1 and Ago4, suggests that our transcriptome assembly does provide a broad representation of expressed genes in *N. benthamiana*. With the exception of Rdr1, which is discussed below, if this plant is defective in any of the RNAi pathways it is not because of the absence of transcription of any of these 31 core genes.

### Assessment of Nb-AGO, Nb-DCL, Nb-DRB and Nb-RDR Genes

**Figure 6:**
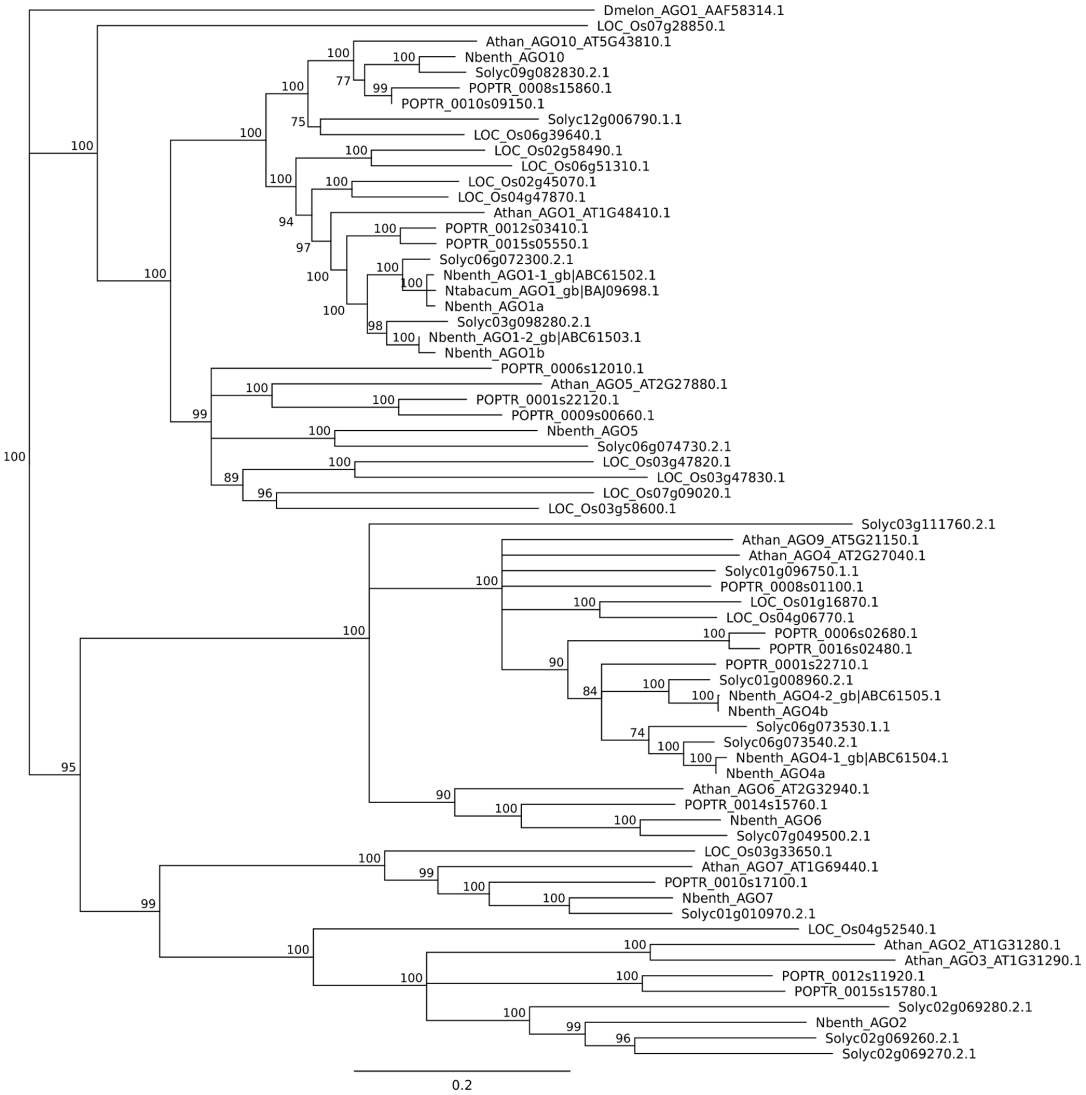
Neighbour joining tree of AGO proteins. Sequences from *N. benthamiana* (Nbenth), *A. thaliana* (Athan), *S. lycopersicum* (Solyc), *O. sativa* (LOC_Os) and *P. trichocarpa* (POPTR) were aligned with the MUSCLE algorithm. Bootstrap values are shown at the nodes. The Athan sequences can be used to classify the AGO family. In all clades, the Nbenth sequences clearly group with Solyc sequences.

The AGO, DCL, DRB and RDR sequences from the *N. benthamiana* transcriptome, along with homologues from *O. sativa*, *P. trichocarpa*, *S. lycopersicum*, *A. thaliana* and some *Nicotiana* species, including previously reported *N. benthamiana* sequences from NCBI, were retrieved from their respective databases and analysed for phylogenetic relationships. Figure 6 shows the neighbour joining tree of AGO sequences (see Figure S3, S4, and S5 for trees of DCLs, DRBs and RDRs). As expected, sequences from the *Nicotiana* species formed their own clades, and are closely related to the *S. lycopersicum* sequences.

**Figure 7:**
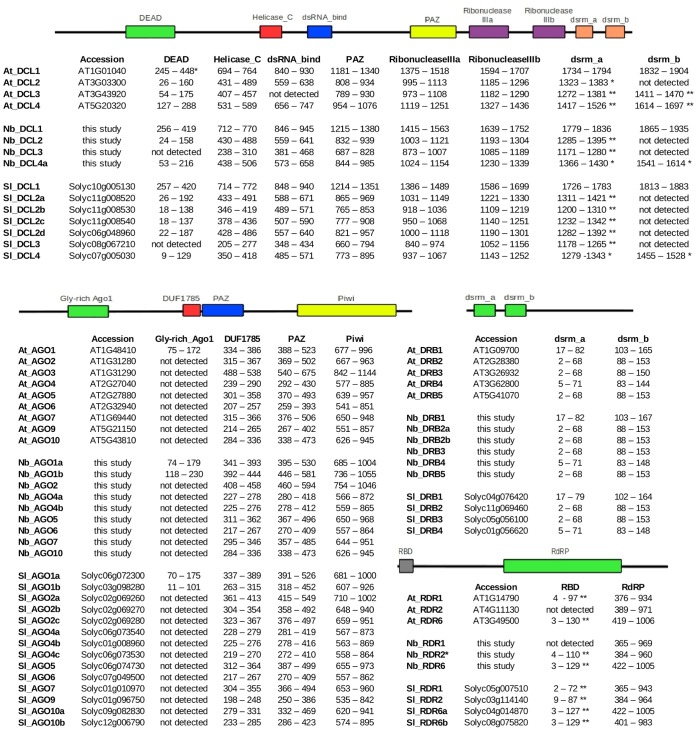
Domains of RNAi proteins. The figure shows the domains of the RNAi proteins AGO, DCL, DRB and RDR from *N. benthamiana* (Nb), *A. thaliana* (At, from TAIR) and *S. lycopersicum* (Sl, from Solgenomics). Domains were detected with InterProScan against all its default databases, and defined according to the Pfam predictions unless otherwise annotated (*according to SMART database; **according to SUPERFAMILY database).

**Figure 8:**
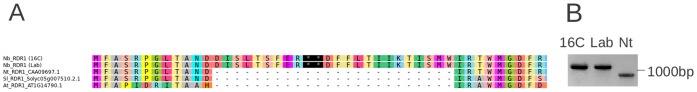
Insertion in the RDR1 sequence of *N. benthamiana*. (A) Alignment of the RDR1 sequence from two lines of *N. benthamiana* (Nb) (16C and Lab), *N. tabacum* (Nt), *S. lycopersicum* (Sl) and *A. thaliana* (At). Only the Nb lines possess an insertion containing two stop codons. (B): PCR of region flanking the 72 base insert in Nb 16C and Lab lines, and Nt, indicating that the insertion is only present in *N. benthamiana*.

To further assess whether the AGO, DCL, DRB and RDR sequences identified in this study conformed to the characteristics representative of each category, they and their *A. thaliana* and *S. lycopersicum* counterparts were searched for protein domains using InterProScan. Most of the characteristic domains for the genes in each category were identified (Figure 7) but there were some notable exceptions. Nb-RDR1 contains two premature stop codons, from a 72bp insertion, within its RdRP domain (Figure 8A), in the first exon of the coding sequence. This insertion has been previously reported [54], [55] and we further verified its existence, in both our lab line and in a well-known GFP-expressing line (16C) [56], [58], using PCR with primers flanking the insertion site (Fig. 8B). Interestingly, PCR indicated the absence of this insertion in *N. tabacum*, and sequence comparison with the RDR of tomato and *A. thaliana* implied that this insertion is so far particular to *N. benthamiana*.

Two other notable exceptions are in Nb-DCL3, which appears to have only one double stranded RNA motif (dsrm) in its C-terminal region and to lack the DEAD motif in its N-terminal helicase region (Figure 7). A tandem pair of dsrms (a and b) in DCL1, DCL3 and DCL4, and a single dsrm in DCL2, seemed to be the canonical arrangement in plants [59]. However, both Nb-DCL3 and Sl-DCL3 lack dsrm-b. While DCL3 has been shown to generate 24 nt siRNAs from transposons to direct heterochromatin modification [60], [61], it also produces siRNAs from viruses and their satellites and, in concert with DCL4 and DCL2, plays a role in repression of replication [62]. *Nicotiana benthamiana* is hyper-susceptible to viruses and it is possible that, besides the inactivation of RDR1 by the 72bp insertion, the loss of dsrm-b and DEAD motif in Nb-DCL3 may also play a role in this. The same motifs are not present in Sl-DCL3 (Figure 7). There is no premature stop codon in Sl-RDR1 (Figure 8), and tomato is susceptible to over 130 different viruses. This raises the possibility that it may not (only) be the RDR1 insertion in *N. benthamiana* but rather the unusual DCL3, or a combination of several such traits that is engendering weaker viral defence.

**Figure 9:**
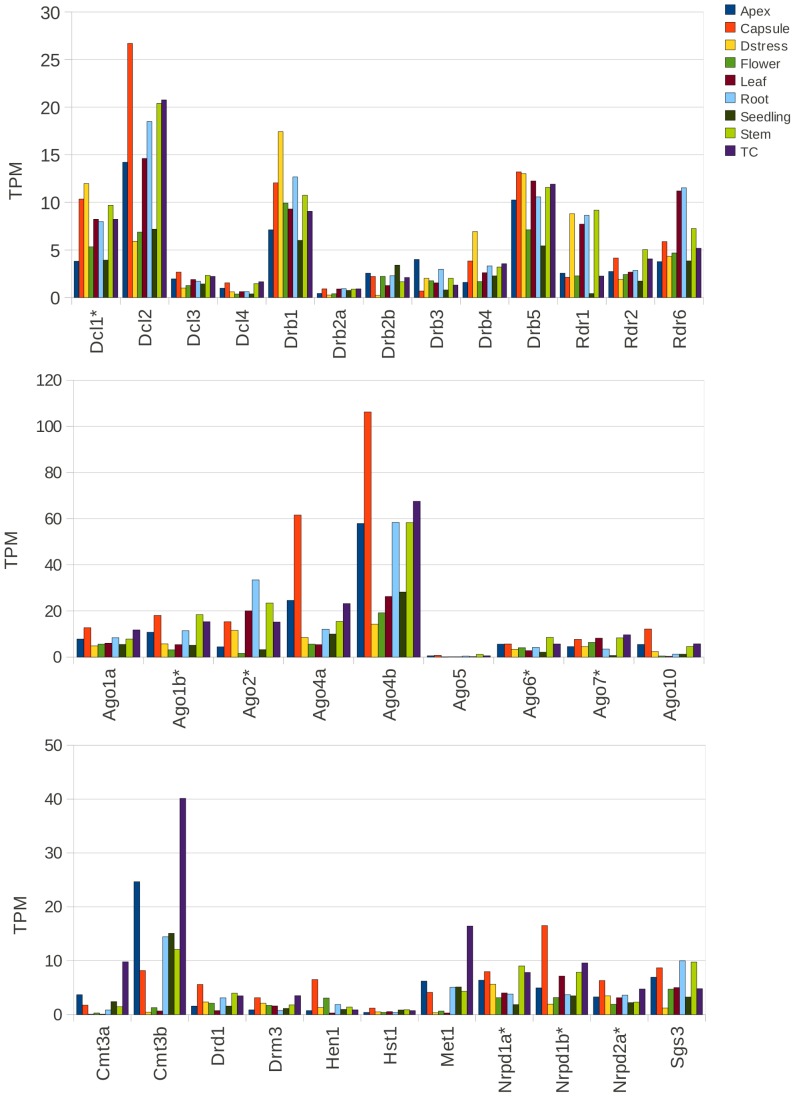
Relative abundances of RNAi-associated genes. The figure shows the relative abundances of RNAi genes identified in this study in terms of TPM (Transcripts Per Million) values calculated from the RSEM software. Genes with an asterisk (*) were comprised of more than oneassembled transcript, and the TPM value in this case represents the TPM sum of all its component transcripts.

In these four gene families, there was a spectrum of expression levels within and across different tissues, as assessed by their TPM values (Figure 9 and Table S5). Overall, Ago4, Dcl1 and 2, Drb1 and 5, and Rdr1 and 6 had higher expression levels than other family members in all tissues. The higher abundance of Dcl1 compared to Dcl3, and Ago4a compared to Ago1a, reported here, does appear to correlate with the findings of other reports [56], [63]. In fact, an RSEM analysis using RNA-seq data from another *N. benthamiana* transcriptome [40] showed a very similar expression profile to our RNAi gene set (Figure S6), supporting the broad-level interpretation of transcript abundances based on our transcriptome.

Ago4a and especially Ago4b transcripts were highly abundant in the apex and capsle tissues. The capsule tissue contains developing seeds and Ago9 has recently been shown to play an important role in transferring the maternal epigenetic overlay to the next generation in *A. thaliana* [64]. Given that AGO4B was most similar to AtAGO9 in our homologue analysis, it is tempting to speculate that this AGO4B might aid similar epigenetic transfer processes in *N. benthamiana*.

Curiously, the level of Nb-DCL4 was relatively low in all tissues whereas the Nb-DCL2 level was very high. This is in contrast to the situation in *A. thaliana*, in which all four DCLs have fairly similar, and relatively low, expression levels. At-DCL4 is the major producer of tasiRNAs and of secondary siRNAs for antiviral activity, whereas At-DCL2 has signalling and back-up anti-viral functions [62], [65]. Perhaps the low expression of NbDCL4 also plays a role in the plant’s susceptibility to viruses. It will be interesting to see how the expression profiles of these genes change upon virus infection.

### Conclusion

We present here the transcriptome of a lab line of *N. benthamiana,* assembled from deep sequencing data from 9 different tissue samples. From comparisons with other databases, it appears to broadly represent the coding capacity of the genome, and should be a useful resource for researchers in the plant community. The annotated transcriptome is available for download and BLAST searches at http://www.benthgenome.com, where a draft genome is also available. The transcriptome has been mapped onto the genome to generate putative gene models, supported by RNA-seq read data of each tissue. These models and mapped reads can be visualised through a Gbrowse portal and may be interrogated by the provided tracks. We also provide on this site the largest list, to date, of *N. benthamiana* RNAi-associated gene sequences, which have been identified bioinformatically from this study. These may be of particular interest to researchers using this plant in RNA silencing studies.

## Supporting Information

Figure S1 **Distribution of GO slim terms.** The figure shows the distribution unigene transcripts from the *de novo N. benthamiana* transcriptome assembly that could be annotated GO slim terms. The distributions are shown as categories of biological process (top panel), cellular component (middle panel), and molecular function (bottom panel). Only terms with more than 10 unigenes are shown.
(TIFF)

Figure S2 **Alignment of previously reported DCL, AGO and RDR sequences against those identified in this study.** The alignment view shows the position of publically available sequences with respect to the sequence identified in this study. The slight difference in sequence identity could be due to differences in *N. benthamiana* lines maintained in different laboratories.
(TIFF)

Figure S3 **Neighbour joining tree of RDR proteins.** Sequences from *N. benthamiana* (Nbenth), *A. thaliana* (Athan), *S. lycopersicum* (Solyc), *O. sativa* (LOC_Os) and *P. trichocarpa* (POPTR) were aligned with the MUSCLE algorithm. Bootstrap values are shown at the nodes.
(TIFF)

Figure S4 **Neighbour joining tree of DRB proteins.** Sequences from *N. benthamiana* (Nbenth), *A. thaliana* (Athan), *S. lycopersicum* (Solyc), *O. sativa* (LOC_Os) and *P. trichocarpa* (POPTR) were aligned with the MUSCLE algorithm. Bootstrap values are shown at the nodes.
(TIFF)

Figure S5 **Neighbour joining tree of DCL proteins.** Sequences from *N. benthamiana* (Nbenth), *A. thaliana* (Athan), *S. lycopersicum* (Solyc), *O. sativa* (LOC_Os) and *P. trichocarpa* (POPTR) were aligned with the MUSCLE algorithm. Bootstrap values are shown at the nodes.
(TIFF)

Figure S6 **Relative abundances of RNAi-associated genes identified in this study using RNA-seq data from** [**40**] **(8DPI dataset).** TPM values were calculated using the RSEM software. With the exception of Drb2b and Rdr1, the overall abundance profile is highly similar to that reported in Figure 9.
(TIFF)

Table S1 **Distribution statistics calculated by the R software (5-number summary) of TPM (top) and normalized EC (bottom) values of transcripts in the 9 tissues used for the transcriptome assembly.**
(DOC)

Table S2 Number of transcripts with normalized EC counts greater than 0 that could be annotated with GO slim terms in each tissue*. *Within the biological process category, biosynthetic process (GO:0009058), cellular nitrogen compound metabolic process (GO:0034641), small molecule metabolic process (GO:0044281), translation (GO:0006412) and transport (GO:0006810) were highly represented in all tissues. Similarly, within the cellular component category, cell (GO:0005623), cytoplasm (GO:0005737), intracellular (GO:0005622) and organelle (GO:0043226) were highly represented. Within the molecular function category, there was no one sub-category that stood out, but oxidoreductase activity (GO:0016491), DNA binding (GO:0003677), ion binding (GO:0043167) and RNA binding (GO:0003723) were noticeably represented categories. The highly similar GO profiles for all tissues may be due to the limited subset of transcripts that could actually be annotated with a GO classification.
(DOC)

Table S3 **Top 10 expressed transcripts (based on EC values) in the 9 tissues used for the transcriptome assembly.**
(DOC)

Table S4 **List of RNAi-associated genes from** ***Arabidopsis thaliana*** **used to screen for orthologues in** ***Nicotiana benthamiana*** **.** 1. Eamens A, Wang M-B, Smith NA, Waterhouse PM (2008) RNA Silencing in Plants: Yesterday, Today, and Tomorrow. Plant Physiol 147: 456–468. 2. Jaubert M, Bhattacharjee S, Mello AFS, Perry KL, Moffett P (2011) ARGONAUTE2 Mediates RNA-Silencing Antiviral Defenses against Potato virus X in *Arabidopsis*. Plant Physiol 156: 1556–1564. 3. Vaucheret H (2008) Plant ARGONAUTES. Trends Plant Sci 13: 350–358. 4. Havecker ER, Wallbridge LM, Hardcastle TJ, Bush MS, Kelly KA, et al. (2010) The *Arabidopsis* RNA-Directed DNA Methylation Argonautes Functionally Diverge Based on Their Expression and Interaction with Target Loci. Plant Cell 22: 321–334. 5. Zhu H, Hu F, Wang R, Zhou X, Sze S-H, et al. (2011) *Arabidopsis* Argonaute10 Specifically Sequesters miR166/165 to Regulate Shoot Apical Meristem Development. Cell 145: 242–256. 6. Eamens AL, Kim KW, Curtin SJ, Waterhouse PM (2012) DRB2 Is Required for MicroRNA Biogenesis in *Arabidopsis thaliana*. PLoS One 7: e35933. 7. Eamens AL, Kim KW, Waterhouse PM (2012) DRB2, DRB3 and DRB5 function in a non-canonical microRNA pathway in *Arabidopsis thaliana*. Plant Signal Behav 7: 1224–1229.
(DOC)

Table S5 **Relative abundances of RNAi-associated genes identified in this study, shown as TPM values calculation by the RSEM software (this is a numerical version of** **Figure 5** **).** *Genes comprised of more than 1 transcript from the assembly; each TPM value represents the sum of all the constituent TPMs.
(DOC)
