# Supplementary figures and images for: De Novo Transcriptome Sequence Assembly and Analysis of RNA Silencing Genes of Nicotiana benthamiana
Source: PLoS One. 2013 Mar 28;8(3):e59534. doi: 10.1371/journal.pone.0059534 (PMC3610648; doi:10.1371/journal.pone.0059534)

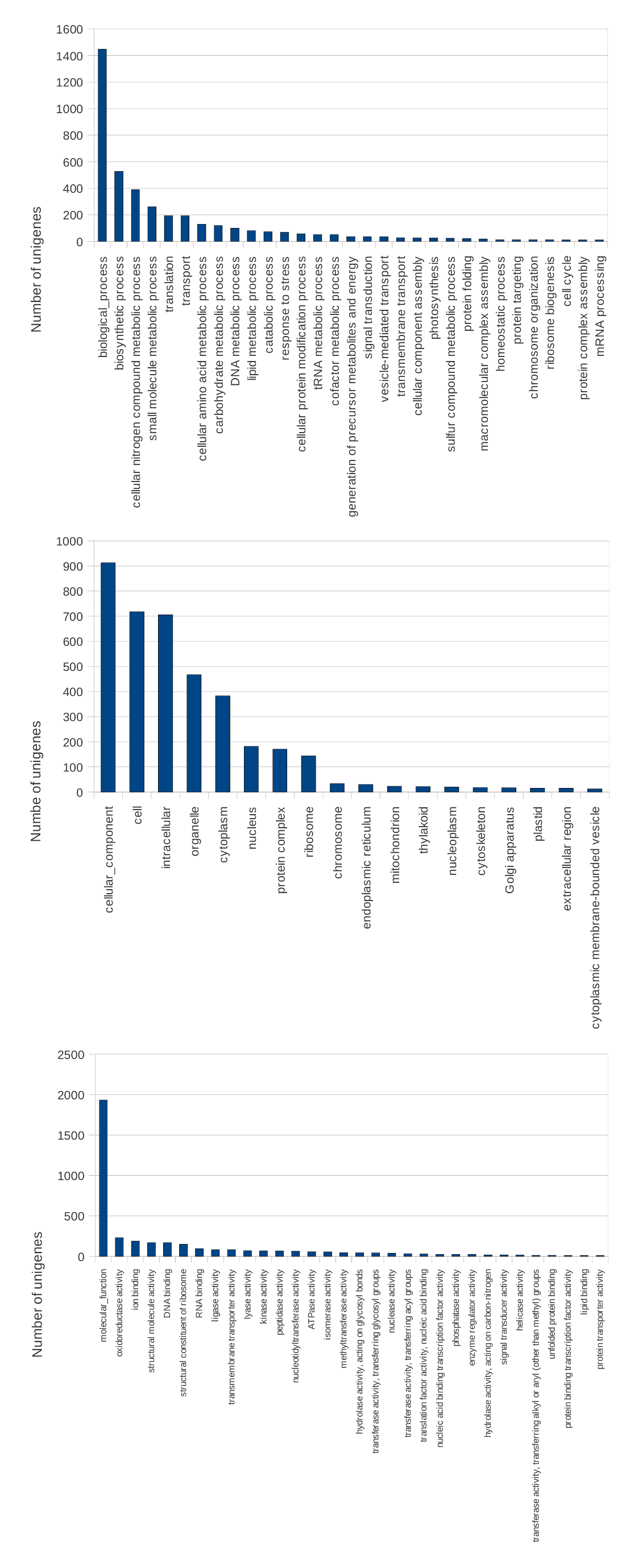

Supplement: Figure S1 — Distribution of GO slim terms. The figure shows the distribution unigene transcripts from the de novo N. benthamiana transcriptome assembly that could be annotated GO slim terms. The distributions are shown as categories of biological process (top panel), cellular component (middle panel), and molecular function (bottom panel). Only terms with more than 10 unigenes are shown. (TIFF) [file pone.0059534.s001.tiff]

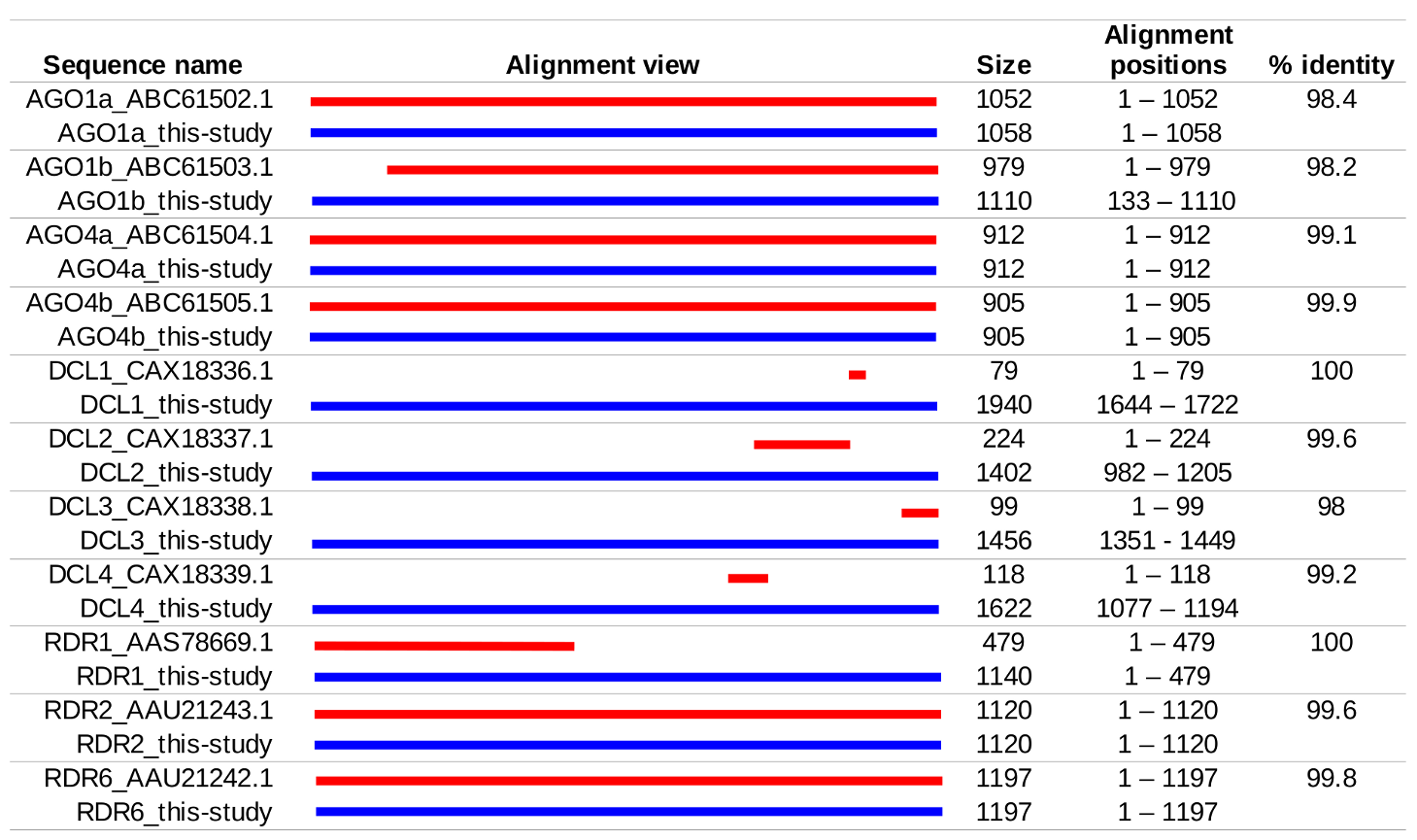

Supplement: Figure S2 — Alignment of previously reported DCL, AGO and RDR sequences against those identified in this study. The alignment view shows the position of publically available sequences with respect to the sequence identified in this study. The slight difference in sequence identity could be due to differences in N. benthamiana lines maintained in different laboratories. (TIFF) [file pone.0059534.s002.tiff]

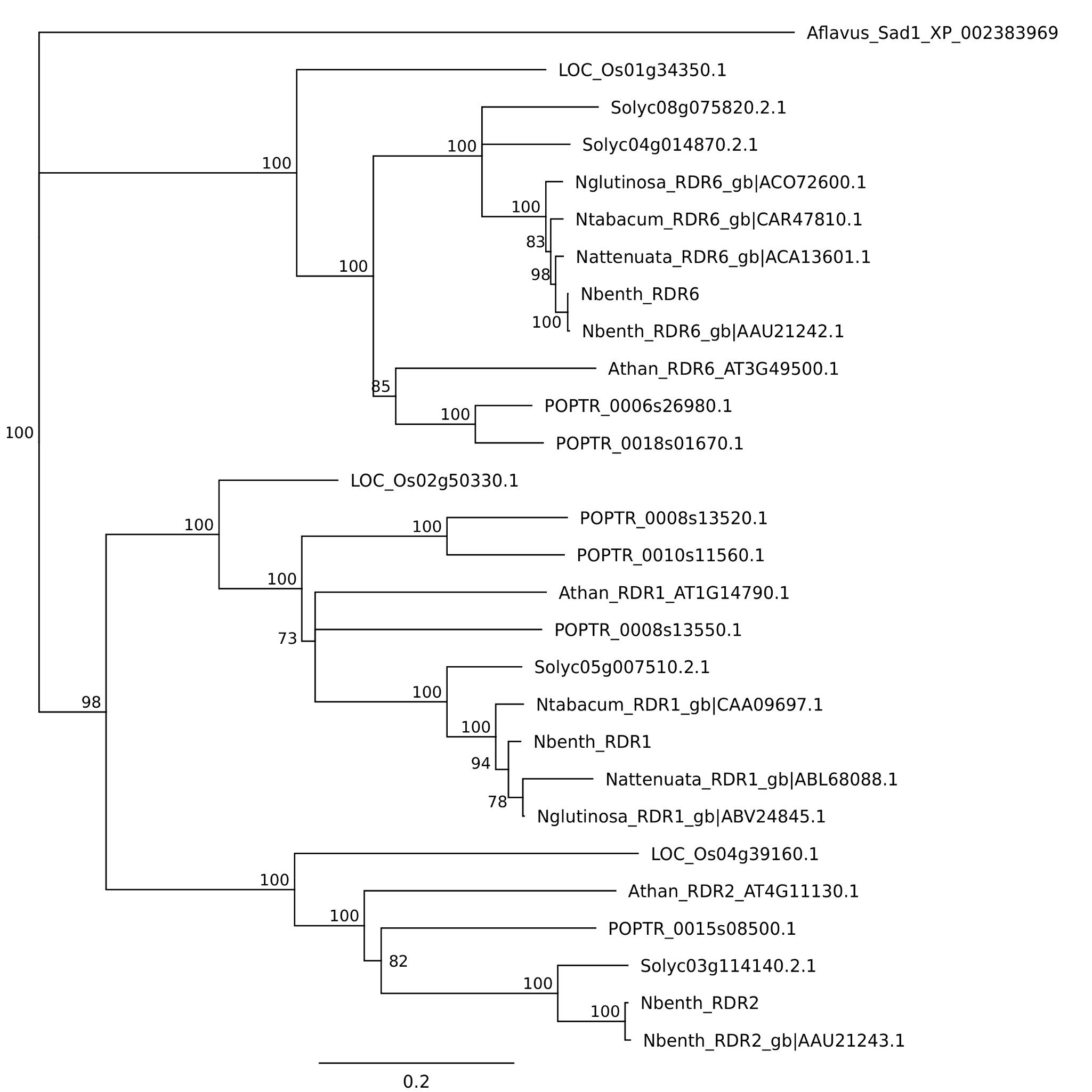

Supplement: Figure S3 — Neighbour joining tree of RDR proteins. Sequences from N. benthamiana (Nbenth), A. thaliana (Athan), S. lycopersicum (Solyc), O. sativa (LOC_Os) and P. trichocarpa (POPTR) were aligned with the MUSCLE algorithm. Bootstrap values are shown at the nodes. (TIFF) [file pone.0059534.s003.tiff]

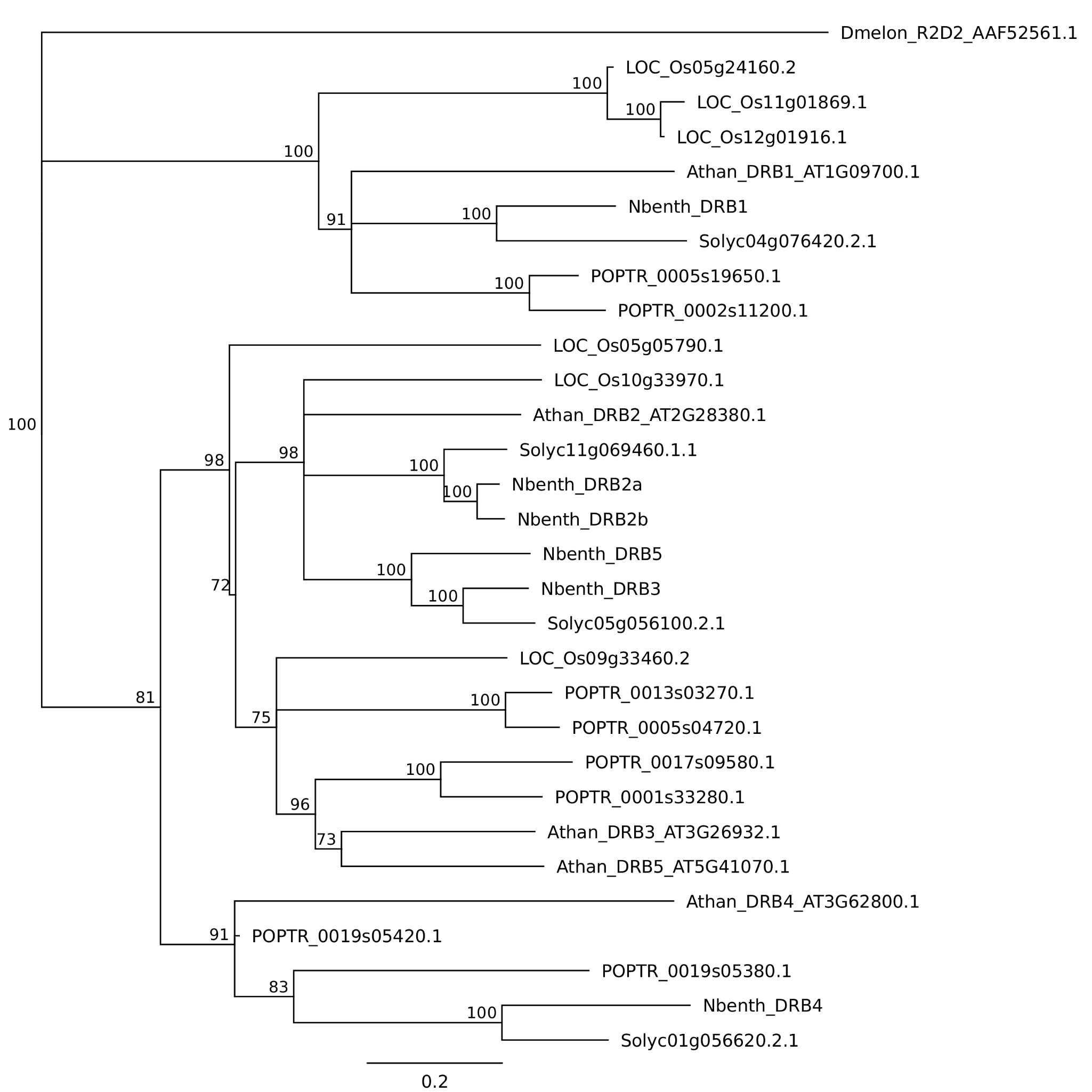

Supplement: Figure S4 — Neighbour joining tree of DRB proteins. Sequences from N. benthamiana (Nbenth), A. thaliana (Athan), S. lycopersicum (Solyc), O. sativa (LOC_Os) and P. trichocarpa (POPTR) were aligned with the MUSCLE algorithm. Bootstrap values are shown at the nodes. (TIFF) [file pone.0059534.s004.tiff]

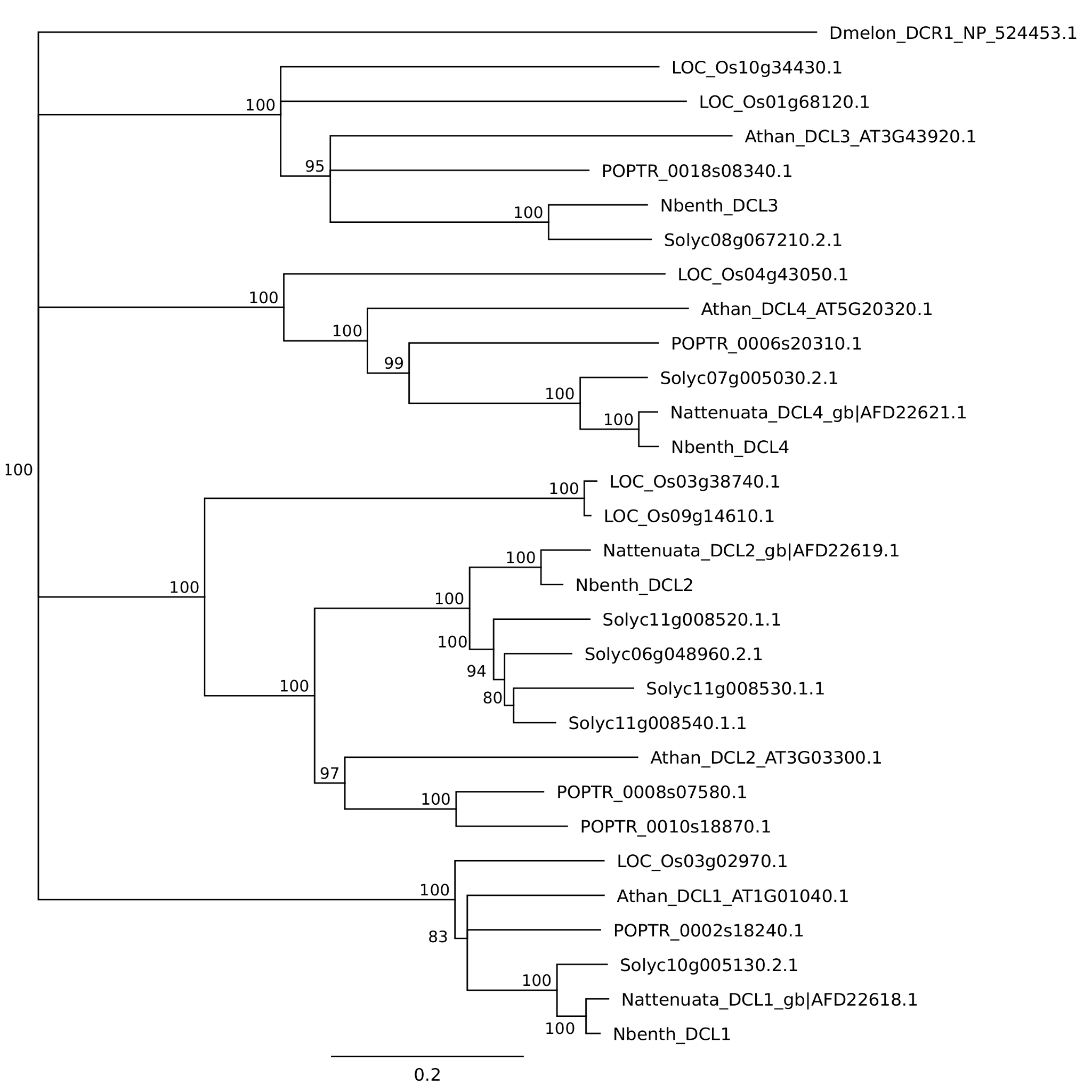

Supplement: Figure S5 — Neighbour joining tree of DCL proteins. Sequences from N. benthamiana (Nbenth), A. thaliana (Athan), S. lycopersicum (Solyc), O. sativa (LOC_Os) and P. trichocarpa (POPTR) were aligned with the MUSCLE algorithm. Bootstrap values are shown at the nodes. (TIFF) [file pone.0059534.s005.tiff]

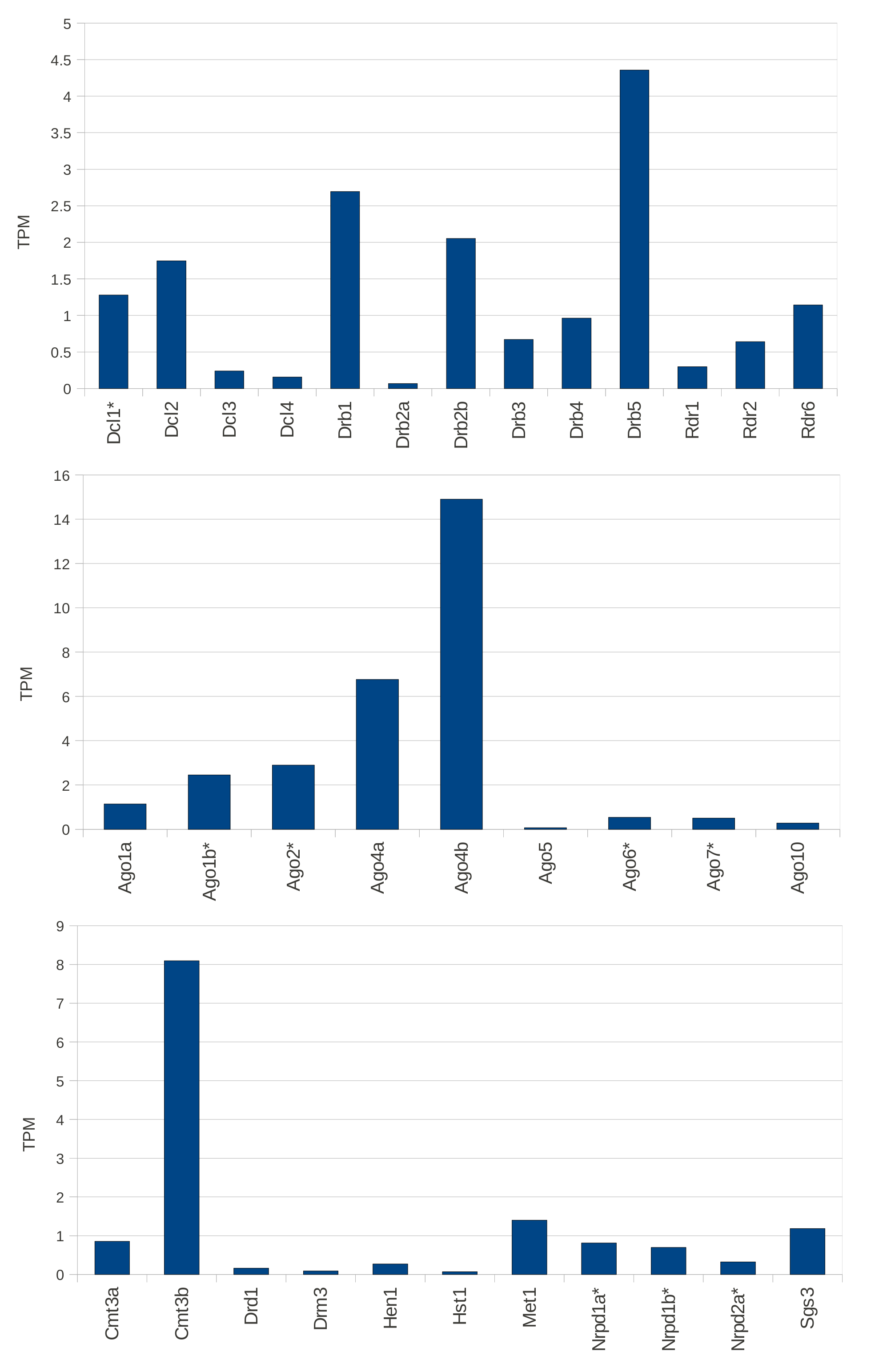

Supplement: Figure S6 — Relative abundances of RNAi-associated genes identified in this study using RNA-seq data from [40] (8DPI dataset). TPM values were calculated using the RSEM software. With the exception of Drb2b and Rdr1, the overall abundance profile is highly similar to that reported in Figure 9. (TIFF) [file pone.0059534.s006.tiff]
