# Supplementary material for: De Novo Transcriptome Sequence Assembly and Analysis of RNA Silencing Genes of Nicotiana benthamiana
Source: PLoS One. 2013 Mar 28;8(3):e59534. doi: 10.1371/journal.pone.0059534 (PMC3610648; doi:10.1371/journal.pone.0059534)
Supplement: Table S4 — List of RNAi-associated genes from Arabidopsis thaliana used to screen for orthologues in Nicotiana benthamiana . 1. Eamens A, Wang M-B, Smith NA, Waterhouse PM (2008) RNA Silencing in Plants: Yesterday, Today, and Tomorrow. Plant Physiol 147: 456–468. 2. Jaubert M, Bhattacharjee S, Mello AFS, Perry KL, Moffett P (2011) ARGONAUTE2 Mediates RNA-Silencing Antiviral Defenses against Potato virus X in Arabidopsis. Plant Physiol 156: 1556–1564. 3. Vaucheret H (2008) Plant ARGONAUTES. Trends Plant Sci 13: 350–358. 4. Havecker ER, Wallbridge LM, Hardcastle TJ, Bush MS, Kelly KA, et al. (2010) The Arabidopsis RNA-Directed DNA Methylation Argonautes Functionally Diverge Based on Their Expression and Interaction with Target Loci. Plant Cell 22: 321–334. 5. Zhu H, Hu F, Wang R, Zhou X, Sze S-H, et al. (2011) Arabidopsis Argonaute10 Specifically Sequesters miR166/165 to Regulate Shoot Apical Meristem Development. Cell 145: 242–256. 6. Eamens AL, Kim KW, Curtin SJ, Waterhouse PM (2012) DRB2 Is Required for MicroRNA Biogenesis in Arabidopsis thaliana. PLoS One 7: e35933. 7. Eamens AL, Kim KW, Waterhouse PM (2012) DRB2, DRB3 and DRB5 function in a non-canonical microRNA pathway in Arabidopsis thaliana. Plant Signal Behav 7: 1224–1229. (DOC) [file pone.0059534.s010.doc]

| **Protein** | **Locus** | **Protein Class/Function** | **Reference** |
| --- | --- | --- | --- |
| AGO1 | AT1G48410 | RNA slicer/core component of plant RISC |  |
| AGO2 | AT1G31280 | RNA slicer/antiviral defense |  |
| AGO3 | AT1G31290 | RNA slicer/highly similar to AGO2; unknown function |  |
| AGO4 | AT2G27040 | RNA slicer/involved in the establishment phase of RdDM |  |
| AGO5 | AT2G27880 | RNA slicer/paralogue of AGO1; unknown function |  |
| AGO6 | AT2G32940 | RNA slicer/tasiRNA-directed heterochromatin formation; RdDM pathway |  |
| AGO7 | AT1G69440 | RNA slicer/tasiRNA biogenesis and juvenile-to-adult transition |  |
| AGO9 | AT5G21150 | RNA slicer/involved in RdDM pathway |  |
| AGO10 | AT5G43810 | RNA slicer/sequestration of specific miRNAs |  |
| CMT3 | AT1G69770 | Methyltransferase/maintenance phase of RdDM |  |
| DCL1 | AT1G01040 | RNase III/miRNA, natsiRNA, and tasiRNA biogenesis |  |
| DCL2 | AT3G03300 | RNase III/natsiRNA biogenesis and viral defense |  |
| DCL3 | AT3G43920 | RNase III/rasiRNA biogenesis and establishment phase of RdDM |  |
| DCL4 | AT5G20320 | RNase III/tasiRNA biogenesis and viral defense |  |
| DRB1 (HYL1) | AT1G09700 | dsRNA binding protein/miRNA and tasiRNA biogenesis |  |
| DRB2 | AT2G28380 | dsRNA binding protein/miRNA biogenesis |  |
| DRB3 | AT3G26932 | dsRNA binding protein/translational repression based silencing |  |
| DRB4 | AT3G62800 | dsRNA binding protein/miRNA and tasiRNA biogenesis |  |
| DRB5 | AT5G41070 | dsRNA binding protein/translational repression based silencing |  |
| DRD1 | AT2G16390 | SNF2-like chromatin-remodeling factor/establishment phase of RdDM |  |
| DRM3 | AT3G17310 | Methyltransferase/establishment phase of RdDM |  |
| HEN1 | AT4G20910 | sRNA-specific methyltransferase/sRNA biogenesis |  |
| HST1 | AT3G05040 | Exportin-5 ortholog/miRNA exportation from nucleus |  |
| MET1 | AT5G49160 | Methyltransferase/maintenance phase of RdDM |  |
| NRPD1a | AT1G63020 | DNA-dependent RNA polymerase/establishment phase of RdDM |  |
| NRPD1b | AT2G40030 | DNA-dependent RNA polymerase/establishment phase of RdDM |  |
| NRPD2 | AT3G23780 | DNA-dependent RNA polymerase/establishment phase of RdDM |  |
| RDR1 | AT1G14790 | RNA-dependent RNA polymerase/viral defense |  |
| RDR2 | AT4G11130 | RNA-dependent RNA polymerase/rasiRNA biogenesis |  |
| RDR6 | AT3G49500 | RNA-dependent RNA polymerase/tasiRNA and natsiRNA biogenesis |  |
| SGS3 | AT5G23570 | Coiled-coil protein/tasiRNA and natsiRNA biogenesis |  |
