# Supplementary material for: De Novo Transcriptome Sequence Assembly and Analysis of RNA Silencing Genes of Nicotiana benthamiana
Source: PLoS One. 2013 Mar 28;8(3):e59534. doi: 10.1371/journal.pone.0059534 (PMC3610648; doi:10.1371/journal.pone.0059534)
Supplement: Supplementary file 3 [file pone.0059534.s007.doc]

| TPM | | | | | | |
|---|---|---|---|---|---|---|
| | **Minimum** | **1st Quartile** | **Median** | **Mean** | **3rd Quartile** | **Maximum** |
| Apex | 0.05 | 1.008 | 1.735 | 4.213 | 2.829 | 21910 |
| Caps | 0.039 | 0.931 | 1.553 | 4.213 | 3.002 | 11960 |
| Dstress | 0.04 | 0.913 | 1.679 | 4.213 | 3.358 | 4158 |
| Flower | 0.043 | 0.765 | 1.316 | 4.213 | 2.423 | 8996 |
| Leaf | 0.0309 | 0.7514 | 1.291 | 4.213 | 2.491 | 3158 |
| Root | 0.0265 | 0.7065 | 1.265 | 4.213 | 2.404 | 1670 |
| Seedl | 0.004 | 0.21 | 0.469 | 4.213 | 1.351 | 3493 |
| Stem | 0.0436 | 0.9983 | 1.664 | 4.213 | 3.171 | 1314 |
| TCC | 0.052 | 1.049 | 1.72 | 4.213 | 3.081 | 2770 |
| **Normalized EC** | | | | | | |
| Apex | 0.01 | 0.66 | 1.98 | 7.05 | 5.74 | 16065 |
| Caps | 0.01 | 0.45 | 1.48 | 6.83 | 5.06 | 24114 |
| Dstress | 0.01 | 0.43 | 1.45 | 6.63 | 4.96 | 4411 |
| Flo | 0.01 | 0.5 | 1.76 | 9.03 | 5.81 | 10491 |
| Leaf | 0.01 | 0.44 | 1.61 | 7.72 | 5.53 | 8270 |
| Roots | 0.01 | 0.46 | 1.68 | 8.62 | 5.95 | 3827 |
| Seedl | 0.01 | 0.25 | 1.13 | 9.56 | 5.24 | 5240 |
| Stem | 0.01 | 0.44 | 1.5 | 6.1 | 5.12 | 2634 |
| TC | 0.01 | 0.5 | 1.78 | 6.58 | 5.71 | 1573 |
