# Supplementary material for: De Novo Transcriptome Sequence Assembly and Analysis of RNA Silencing Genes of Nicotiana benthamiana
Source: PLoS One. 2013 Mar 28;8(3):e59534. doi: 10.1371/journal.pone.0059534 (PMC3610648; doi:10.1371/journal.pone.0059534)
Supplement: Supplementary file 4 [file pone.0059534.s008.doc]

	Apex	Caps	Dstress	Flower	Leaf	Root	Seedl	Stem	TC
Biological process	4794	4890	4791	4879	4845	4849	5064	4822	4912
Cellular component	4309	4369	4301	4356	4365	4318	4468	4387	4360
Molecular function	4344	4399	4327	4396	4383	4369	4496	4365	4393
