# Supplementary material for: De Novo Transcriptome Sequence Assembly and Analysis of RNA Silencing Genes of Nicotiana benthamiana
Source: PLoS One. 2013 Mar 28;8(3):e59534. doi: 10.1371/journal.pone.0059534 (PMC3610648; doi:10.1371/journal.pone.0059534)
Supplement: Supplementary file 5 [file pone.0059534.s009.doc]

	Description	Normalized ECs		Description	Normalized Ecs
**Apex**			**Root**		
Niv3K765635181	Protein TAP1 precursor	16064.67	Niv3K805659615	Tubulin alpha chain	3827.21
Niv3K765636475	Defensin-like protein, Precursor	7269.61	Niv3K605747902	Glucan endo-1,3-beta-glucosidase, basic vacuolar isoform GGIB50 ((1->3)-beta-glucanase), Precursor	2109.21
Niv3K725839522	Wound-induced proteinase inhibitor 2, Precursor	4634.68	Niv3K725610051	Catalase isozyme 1 (SABP)	1865.47
Niv3K805655645	Ethylene-responsive proteinase inhibitor 1, Precursor	4468.43	Niv3K745621433	5-methyltetrahydropteroyltriglutamate--homocysteine methyltransferase	1755.91
Niv3K785653169	Non-specific lipid-transfer protein (LTP), Precursor	3021.19	Niv3K765637499	Phosphatidylserine decarboxylase beta chain	1589.98
Niv3K765632833	serine protease inhibitor 2	1987.24	Niv3K725613519	Quinolinate synthase A	1292.89
Niv3K805659615	Tubulin alpha chain	1381.23	Niv3K665800008	Probable glutathione S-transferase	1272.6
Niv3K725843175	Carbonic anhydrase, chloroplastic, Precursor	1220.92	Niv3K725612271	Ubiquitin, Precursor	1171.9
Niv3K805656915	TRAF2 and NCK-interacting protein kinase	1119.03	Niv3K805659034	Pleiotropic drug resistance protein 1	1150.17
Niv3K805655807	Sugar transport protein 8	1076.23	Niv3K585694414	Linoleate 9S-lipoxygenase 6 (StLOX1)	1138.57
**Capsule**			**Seedling**		
Niv3K805662064	11S globulin seed storage protein 2 basic chain, Precursor	24113.92	Niv3K805659615	Tubulin alpha chain	5239.54
Niv3K785649564	Legumin B basic chain, Precursor	12178.28	Niv3K805656982	chlorophyll a-b binding protein chloroplastic	5116.42
Niv3K705825953	11S globulin delta chain, Precursor	10739.24	Niv3K805657150	chlorophyll a-b binding protein chloroplastic	4494.81
Niv3K805658849	11S globulin seed storage protein G3 basic chain, Precursor	9847.06	Niv3K805658200	chlorophyll a-b binding protein chloroplastic	4443.89
Niv3K625771317	Legumin B basic chain, Precursor	9116.33	Niv3K805658276	chlorophyll a-b binding protein chloroplastic	4285.47
Niv3K785648979	Legumin B basic chain, Precursor	7362.68	Niv3K785645902	Probable aquaporin PIP-type pTOM75 (RAMP)	3652.84
Niv3K805657565	Glutelin type-B 4 basic chain, Precursor	5926.42	Niv3K805657057	Photosystem I reaction center subunit II, chloroplastic (PSI-D), Precursor	3466.95
Niv3K705821994	2S albumin large chain, Precursor	5601.14	Niv3K765638032	chlorophyll a-b binding protein chloroplastic	3299.4
Niv3K785648717	11S globulin seed storage protein 2 basic chain, Precursor	5107.34	Niv3K785650107	chlorophyll a-b binding protein chloroplastic	3248.94
Niv3K585717150	Legumin B basic chain, Precursor	4833.52	Niv3K805657569	chlorophyll a-b binding protein chloroplastic	3186.63
**Dstress**			**Stem**		
Niv3K805657258	Vignain, Precursor	4411.41	Niv3K805659615	Tubulin alpha chain	2633.54
Niv3K785646208	Retrotransposon-derived protein PEG10 (MyEF-3)	2872.11	Niv3K805656914	Ribulose bisphosphate carboxylase/oxygenase activase 1, chloroplastic (RA 1), Precursor	1032.01
Niv3K785647827	Vignain, Precursor	1895.37	Niv3K785649838	Chitinase-like protein 1 (AtCTL1), Precursor	981.21
Niv3K765642608	S-adenosylmethionine decarboxylase beta chain, Precursor	1792.66	Niv3K785652518	Sucrose synthase	945.22
Niv3K785647202	Kirola	1653.07	Niv3K785652507	Glycine dehydrogenase [decarboxylating], mitochondrial, Precursor	932.09
Niv3K805664518	Cysteine proteinase RD21a (RD21), Precursor	1648.31	Niv3K785651961	Ubiquitin, Precursor	931.83
Niv3K785645226	Capsid protein (CP)	1626.28	Niv3K725610051	Catalase isozyme 1 (SABP)	928.44
Niv3K805662978	E3 ubiquitin ligase interacting with arginine methyltransferase (ISS)	1612.48	Niv3K745621433	5-methyltetrahydropteroyltriglutamate--homocysteine methyltransferase	926.05
Niv3K765638189	Vacuolar-processing enzyme (VPE), Precursor	1597.97	Niv3K785648843	Heat shock cognate 70 kDa protein 2	802.47
Niv3K785646029	S-adenosylmethionine decarboxylase beta chain, Precursor	1428.33	Niv3K725612271	Ubiquitin, Precursor	801.73
**Flower**			**TC**		
Niv3K805658722	Heme A synthase (HAS)	10491.01	Niv3K805659615	Tubulin alpha chain	1572.53
Niv3K805656915	TRAF2 and NCK-interacting protein kinase	7690.8	Niv3K745627590	Polyphenol oxidase E, chloroplastic (PPO), Precursor	1486.26
Niv3K805655807	Sugar transport protein 8	7388	Niv3K605747902	Glucan endo-1,3-beta-glucosidase, basic vacuolar isoform GGIB50 ((1->3)-beta-glucanase), Precursor	1439.16
Niv3K805662029	Putative uncharacterized protein ART2	6279.62	Niv3K785645536	Pectinesterase U1 (PE U1), Precursor	1057.07
Niv3K765638243	Salicylate O-methyltransferase (CbSAMT)	4510.06	Niv3K765634716	Elongation factor 1-alpha (EF-1-alpha)	914.22
Niv3K725610051	Catalase isozyme 1 (SABP)	4200.6	Niv3K785645902	Probable aquaporin PIP-type pTOM75 (RAMP)	835.41
Niv3K725840485	Bidirectional sugar transporter SWEET5 (AtSWEET5)	3098.11	Niv3K725610051	Catalase isozyme 1 (SABP)	831.38
Niv3K805661757	Putative uncharacterized protein YLR154W-F	2893.75	Niv3K765636476	Elongation factor 1-alpha (EF-1-alpha)	780.5
Niv3K805664518	Cysteine proteinase RD21a (RD21), Precursor	2587.21	Niv3K725612271	Ubiquitin, Precursor	764.28
Niv3K805662548	Protein YLR162W	2555.26	Niv3K805658722	Heme A synthase (HAS)	756.73
**Leaf**					
Niv3K725612375	Uncharacterized protein ORF91	8269.79			
Niv3K805656915	TRAF2 and NCK-interacting protein kinase	2531.08			
Niv3K805662029	Putative uncharacterized protein ART2	1992.29			
Niv3K805655994	Glyceraldehyde-3-phosphate dehydrogenase A,	1877.79			
Niv3K805658722	Heme A synthase (HAS)	1797.92			
Niv3K805657897	Ribulose bisphosphate carboxylase/oxygenase activase 2, chloroplastic (RA 2), Precursor	1765.46			
Niv3K805656791	Prenyl transferase	1595.44			
Niv3K785652507	Glycine dehydrogenase [decarboxylating], mitochondrial, Precursor	1471.9			
Niv3K785644426	p6-gag	1407.16			
Niv3K805656914	Ribulose bisphosphate carboxylase/oxygenase activase 1, chloroplastic (RA 1), Precursor	1266.17			
