# Supplementary material for: De Novo Transcriptome Sequence Assembly and Analysis of RNA Silencing Genes of Nicotiana benthamiana
Source: PLoS One. 2013 Mar 28;8(3):e59534. doi: 10.1371/journal.pone.0059534 (PMC3610648; doi:10.1371/journal.pone.0059534)
Supplement: Supplementary file 6 [file pone.0059534.s011.doc]

RNAi gene	Transcript Ids	Apex	Caps	Dstress	Flower	Leaf	Root	Seedl	Stem	TC
Ago1a	Nbv3K705826800	7.77	12.68	4.83	5.62	6.00	8.38	5.42	7.82	11.71
Ago1b*	Nbv3K785652119	10.72	18.02	5.75	3.11	5.41	11.40	5.15	18.43	15.29
	Nbv3K785652117									
	Nbv3K705830082									
Ago2*	Nbv3K585706870	4.41	15.33	11.58	1.58	20.04	33.42	3.16	23.37	15.15
	Nbv3K585676062									
Ago4a	Nbv3K585737054	24.57	61.60	8.44	5.59	5.37	12.07	9.97	15.43	23.16
Ago4b	Nbv3K745626388	57.85	106.26	14.24	19.14	26.28	58.34	28.19	58.27	67.50
Ago5	Nbv3K585731374	0.51	0.74	0.07	0.15	0.10	0.41	0.18	1.14	0.51
Ago6*	Nbv3K705827462	5.58	5.68	3.37	4.03	2.81	4.19	2.09	8.55	5.66
	Nbv3K645784811									
	Nbv3K645784061									
	Nbv3K585708242									
Ago7*	Nbv3K585720936	4.48	7.67	4.47	6.37	8.20	3.46	0.69	8.29	9.60
	Nbv3K585735851									
	Nbv3K585719994									
	Nbv3K665804949									
Ago10	Nbv3K585734208	5.45	12.14	2.37	0.46	0.34	1.20	1.21	4.55	5.76
Cmt3a	Nbv3K625768297	3.69	1.74	0.07	0.27	0.07	0.83	2.39	1.45	9.78
Cmt3b	Nbv3K585704505	24.66	8.16	0.42	1.27	0.65	14.44	15.07	12.09	40.13
Dcl1*	Nbv3K605750463	3.81	10.35	11.97	5.33	8.23	7.98	3.92	9.68	8.22
	Nbv3K745628138									
	Nbv3K585722110									
Dcl2	Nbv3K725833766	14.22	26.69	5.91	6.86	14.61	18.50	7.18	20.40	20.77
Dcl3	Nbv3K585704110	1.95	2.66	0.99	1.25	1.89	1.70	1.43	2.33	2.22
Dcl4	Nbv3K625768999	0.99	1.53	0.60	0.37	0.61	0.61	0.38	1.46	1.66
Drb1	Nbv3K605753726	7.11	12.04	17.43	9.91	9.31	12.67	6.00	10.75	9.05
Drb2a	Nbv3K585718488	0.42	0.91	0.27	0.39	0.89	0.92	0.74	0.88	0.91
Drb2b	Nbv3K605753084	2.54	2.20	0.24	2.21	1.27	2.29	3.39	1.66	2.11
Drb3	Nbv3K725608215	4.00	0.67	2.03	1.76	1.55	2.97	0.79	2.01	1.31
Drb4	Nbv3K725839976	1.59	3.84	6.93	1.67	2.60	3.32	2.26	3.21	3.54
Drb5	Nbv3K585684100	10.25	13.20	13.02	7.12	12.23	10.55	5.42	11.58	11.90
Drd1	Nbv3K585726931	1.57	5.59	2.32	2.09	0.73	3.09	1.57	3.96	3.46
Drm3	Nbv3K745624408	0.85	3.13	2.08	1.67	1.61	0.74	1.13	1.77	3.51
Hen1	Nbv3K645785075	0.72	6.48	1.30	3.07	0.28	1.85	0.96	1.39	0.86
Hst1	Nbv3K625768898	0.40	1.20	0.49	0.40	0.54	0.37	0.83	0.89	0.72
Met1	Nbv3K585692501	6.20	4.12	0.33	0.64	0.28	5.05	5.10	4.33	16.41
Nrpd1a*	Nbv3K585708997	6.36	7.97	5.60	3.11	4.01	3.79	1.82	9.01	7.81
	Nbv3K585738556									
	Nbv3K585698167									
Nrpd1b*	Nbv3K685818512	4.93	16.51	1.93	3.16	7.12	3.66	3.48	7.83	9.57
	Nbv3K685816071									
	Nbv3K585729430									
	Nbv3K585720083									
Nrpd2a*	Nbv3K585720667	3.27	6.32	3.47	1.88	3.13	3.58	2.21	2.29	4.74
	Nbv3K685813559									
Rdr1	Nbv3K745620210	2.56	2.12	8.80	2.27	7.71	8.64	0.41	9.17	2.25
Rdr2	Nbv3K625766705	2.73	4.15	1.91	2.43	2.66	2.85	1.72	5.03	4.06
Rdr6	Nbv3K585707928	3.77	5.86	4.32	4.67	11.19	11.52	3.85	7.24	5.16
Sgs3	Nbv3K785651293	6.92	8.65	1.22	4.71	5.00	9.98	3.23	9.76	4.79
